# Potential Therapeutic Properties of *Olea europaea* Leaves from Selected Cultivars Based on Their Mineral and Organic Profiles

**DOI:** 10.3390/ph17030274

**Published:** 2024-02-22

**Authors:** Natália M. de Oliveira, Jorge Machado, Maria Helena Chéu, Lara Lopes, M. Fátima Barroso, Aurora Silva, Sara Sousa, Valentina F. Domingues, Clara Grosso

**Affiliations:** 1 ICBAS, Laboratory of Applied Physiology, School of Medicine and Biomedical Sciences, University of Porto, 4050-313 Porto, Portugal; 2 CBScin, Centre of Biosciences in Integrative Health, 4250-105 Porto, Portugal; 3 Insight: Piaget Research Center for Ecological Human Development, Instituto Piaget—ISEIT, Estrada do Alto Gaio, 3515-776 Lordosa Viseu, Portugal; 4 REQUIMTE/LAQV, ISEP, Polytechnic of Porto, Rua Dr. António Bernardino de Almeida 431, 4249-015 Porto, Portugal

**Keywords:** olive tree leaves, mineral and organic composition, therapeutic properties

## Abstract

Olive leaves are consumed as an extract or as a whole herbal powder with several potential therapeutic benefits attributed to polyphenols, tocopherol’s isomers, and flavonoids, among others. This study assessed the potential variance in the functional features presented by olive leaves from three different Portuguese cultivars—Cobrançosa, Madural, and Verdeal—randomly mix-cultivated in the geographical area of Vale de Salgueiros. Inorganic analysis determined their mineral profiles while an organic analysis measured their total phenolic and flavonoid content, and scanned their phenolic and tocopherol and fatty acid composition. The extracts’ biological activity was tested by determining their antimicrobial and antioxidant power as well as their ability to inhibit acetylcholinesterase, butyrylcholinesterase, MAO-A/B, and angiotensin-I-converting enzyme. The inorganic profiles showed them to be an inexpensive source able to address different mineral deficiencies. All cultivars appear to have potential for use as possible antioxidants and future alternative antibiotics against some multidrug-resistant microorganisms, with caution regarding the arsenic content in the Verdeal cultivar. Madural’s extract displayed properties to be considered a natural multitarget treatment for Alzheimer’s and Parkinson’s diseases, depression, and cardiometabolic and dual activity for blood pressure modulation. This work indicates that randomly cultivating different cultivars significantly modifies the leaves’ composition while keeping their multifaceted therapeutic value.

## 1. Introduction

*Olea europaea* L. is a small tree of the family Oleaceae, typical in tropical and warm temperate regions. This tree is well known for its olives and occupies a prominent position in the Mediterranean commerce as a prime source of olive oil [1]. The olive tree is native to the Middle East and to the coastal areas of the eastern Mediterranean Basin, being distributed also in western Asia and northern Africa. Although olives are now cultivated in several parts of the world, the Mediterranean region still functions as the major contributor, accounting for about 98% of the world’s olive cultivation [2]. According to archaeological records, olives were commercially explored by the Minoans in Crete as far back as 3000 BC [3]. Ancient Greek literature reveals the use of olive oil for body health and the olive tree has a long history of medicinal and nutritional value. Economically, olives appear as an important good as they yield a nourishing edible oil with potential medicinal functions [4]. The rising awareness about the valuable effects of prime nutrition, including functional foods, the pursuit for olives, and their subproducts, has notably increased in high-income countries with rapid expansion of olive-based products [5,6]. Olive oil is the main dietary fat in the Mediterranean diet, associated with a reduced incidence of cardiovascular diseases and some types of cancers [7,8,9,10]. The beneficial effects of olive oil are mainly attributed to its high content of monounsaturated fatty acids (MUFAs) and multifunctional bioactives including tocopherols, carotenoids, and phenolics [11,12]. In addition, such compounds as oleuropein give the unique flavor and taste of olive oil. The content of these components in olives and olive leaves change according to several factors such as type of cultivar, ripeness, harvesting procedure, and agroclimatic circumstances, as well as the processing methods [13,14]. Through history, olive leaf extracts have attended many uses, from tissue conservation in Ancient Egypt to promoting health as a tonic to treat fever and some tropical diseases such as malaria in the Middle East and Mediterranean Basin [15]. Lately, olive leaves have become part of many diets in the form of an extract or as a whole herbal powder [16,17,18]. Olive leaves’ biological compounds yield cardiovascular-protective properties [19]. Not only do numerous studies confirm that olive leaves are able to decline blood pressure, improve blood flow in the coronary arteries [20,21], regulate arrhythmia, and prevent intestinal smooth muscle spasms [22], but their antimicrobial potential against bacteria, fungi, and mycoplasma has also been highlighted [23,24,25,26,27]. Like olive oil, the therapeutic benefits of *O. europaea L. folium* have been credited to low-molecular-weight polyphenols such as oleuropein (up to 60–90 mg/g dry leaf weight), hydroxytyrosol, tyrosol, and vitamin E isomers of tocopherol, elenolic acid derivatives, caffeic acid, *p*-coumaric acid, and vanillic acid, as well as flavonoids—luteolin, diosmetin, rutin, luteolin-7-glucoside, apigenin-7-glucoside, and diosmetin-7-glucoside [28,29]. The relevance of the whole olive leaf and its extract has been quickly rising in both pharmaceutical and food industries, not only as a functional food but also as a material used in food additives for preservation [30,31]. The main objective of the present work is to verify if randomly mix-cultivating distinct varieties of this plant induces changes in the nutritional, biochemical, and functional leaf features compared to separated cultivation of the selected Portuguese cultivars, and if so, to provide further information about the influence of the type of cultivation and geographic position on the composition of high-value bioactives and its impact on their therapeutic properties.

## 2. Results

### 2.1. Mineral Analysis of the Selected Cultivars

A mineral analysis of aqueous extracts of *O. europaea* Cobrançosa, *O. europaea* Madural, and *O. europaea* Verdeal was made by screening the presence of 18 elements through inductively coupled plasma mass spectroscopy (ICP-MS). It was determined that *O. europaea* Cobrançosa’s mineral content follows a waning predominance of Ca (11 ± 0.05 g/Kg), K, S, P, Mg, Na, Fe, Sr, and Mn (47 ± 0.05 mg/kg) (Table 1). *O. europaea* Madural’s leaves were found to have a predominance in the decreasing order of Ca (12 ± 0.05 g/Kg), K, Mg, P, S, Na, Sr, Fe, Mn, and Zn (17 ± 0.05 mg/kg) (Table 1). Finally, *O. europaea* Verdeal’s leaves displayed a waning order predominance as follows: K (13 g/Kg), Ca, P=S, Mg, Na, Sr, Fe, Mn, Zn (17 ± 0.05 mg/kg) (Table 1). According to Table 1, the major mineral components present in these cultivars are calcium (Ca), potassium (K), phosphorus (P), sulfur (S), and sodium (Na); Ca occupies the higher ranking for *O. europaea* Cobrançosa and Verdeal, whereas K comes first in *O. europaea* Madural. The third and fourth most-prominent positions vary between S and P in *O. europaea* Cobrançosa and Verdeal compared to Mg and P in *O. europaea* Madural. The fifth position is occupied by Mg in *O. europaea* Cobrançosa and Verdeal, while in *O. europaea* Madural, it is occupied by S. *O. europaea* Verdeal leaves also showed a double content of As (7 ± 0.03 mg/kg) (Table 1) compared to *O. europaea* Cobrançosa and Madural (<3.0 ± 0.03 mg/kg) (Table 1). Overall, the mineral composition for *O. europaea* Cobrançosa cultivar is more similar to *O. europaea* Verdeal than to *O. europaea* Madural.

### 2.2. Organic Analysis

#### 2.2.1. Nutritional Assessment of OLEs from Selected Cultivars

The water content of the leaves of *O. europaea* Cobrançosa, *O. europaea* Madural, and *O. europaea* Verdeal was determined from aqueous extracts through infrared hygrometry readings. The proximate compositions of the three varieties of samples were verified according to AOAC (2000, 2005, 2006) methods [32,33,34,35], drying the milled leaves at 105 °C to constant weight. Ash weight was determined via incineration at 500 °C. Total protein quantification was measured using the Kjeldahl method, while the total lipids were quantified according to Folch et al., 1957 and using Folch solution. The nutritional assessment of both the *O. europaea* Cobrançosa and Madural cultivars showed a predominance of carbohydrate > protein > lipid > ash. As for the *O. europaea* Verdeal leaves, the predominance was as follows: carbohydrate > lipid > ash > protein. TFC showed a higher presence among the cultivars in the following order: *O. europaea* Verdeal > *O. europaea* Cobrançosa > *O. europaea* Madural. The higher nutritional content in all of the cultivars was water (within the moisture parameter), whose analysis displayed very close results between the three olive leaf extracts (OLEs), with a very-slightly higher value for *O. europaea* Madural. The protein content showed similar results for *O. europaea* Cobrançosa and Madural, while in the *O. europaea* Verdeal leaves, this value falls to approximately half, about 3.81 ± 0.02% (Table 2). The lipidic composition was higher for the *O. europaea* Cobrançosa leaf extract, followed by the *O. europaea* Verdeal and Madural leaf extracts, with this content being very similar between them. Very much alike to the lipidic element, the results for the ash content showed similar values for all of the cultivars, with a slightly higher value for *O. europaea* Verdeal.

#### 2.2.2. Phenolic Composition of OLEs from Selected Cultivars

The phenolic content of hydroethanolic extracts of the leaves from *O. europaea* Cobrançosa, *O. europaea* Verdeal, and *O. europaea* Madural was measured via a colorimetric assay based on Folin–Ciocalteu reagent. It showed a relative higher presence in the following order: *O. europaea* Madural > *O. europaea* Verdeal> *O. europaea* Cobrançosa. The phenolic profile was also studied with HPLC-DAD. The leaves of *O. europaea* Cobrançosa displayed a phenolic differential composition following a waning order of hydroxytyrosol > apigenin derivative > apigenin-7-*O*-glucoside > luteolin-7-*O*-glucoside > verbascoside. The leaves of the *O. europaea* Madural cultivar also show a phenolic composition very close to *O. europaea* Cobrançosa, switching only the third position for apigenin-7-*O*-glucoside and the fourth position to apigenin-7-*O*-glucoside. The leaves of the *O. europaea* Verdeal cultivar showed a slight distinct phenolic arrangement compared to both the previous cultivars, with hydroxytyrosol standing as the major compound followed by apigenin derivative > luteolin-7-*O*-glucoside > verbascoside > apigenin-7-*O*-glucoside. Both *O. europaea* Madural’s and Verdeal’s extracts displayed a higher content of HT: 10.86 ± 0.88 and 10.64 ± 0.15 mg/g dried extract, respectively (Table 3), accounting for 38.76% and 46.12% of the whole phenolic composition, respectively (Table 3)For all three studied cultivars, the least-present phenolic compounds were both caffeic acid and tyrosol.

#### 2.2.3. Fatty Acid Composition of the OLEs of Selected Cultivars

The assessment of the whole fatty acid content of the hydroethanolic extracts from *O. europaea* Cobrançosa, *O. europaea* Verdeal, and *O. europaea* Madural, through gas chromatography analyses, showed overall similar total compositions amongst the three cultivars. Profiling the relative FA content, the results show Σ saturated fatty acids (SFAs) > Σ monounsaturated fatty acids (MUFAs) > Σ polyunsaturated fatty acids (PUFAs) at percentages of 48.6%, 39.9%, and 11.5% for *O. europaea* Cobrançosa; 46.71%, 37.87%, and 15.42% for *O. europaea* Madural; and finally 45.75%, 39.74%, and 14.51% for *O. europaea* Verdeal, respectively (Table 4). The analysis of saturated fatty acids showed a relative higher composition for all three cultivars as follows: palmitic acid (C16:0) > stearic acid (C18:0) > lignoceric acid (C24:0), from which stearic acid was about 25% of the palmitic acid value for all cultivars; lignoreric acid was about 6% of palmitic acid for *O. europaea* Cobrançosa cultivar, whereas it was 10% for both *O. europaea* Madural and Verdeal. The most prominent MUFA for all cultivars was oleic acid (OA) (C18:1n-9c) > cis-11-eicoisanoic acid (C20:1n-9)—the latter accounted for about 50% of OA for *O. europaea* Cobrançosa; around 81% for *O. europaea* Verdeal; and around 88% for *O. europaea* Madural (Table 4). Regarding PUFAs, the total amount of omega-6 fatty acids (FAs) appeared to be higher above the total number of omega-3 FAs regardless of the cultivar. The results of the PUFA profiling were alike for all samples, as follows: linoleic acid (LO) (C18:2 n-6c) > gamma linolenic acid (γ-LA) (C18:3 n-6) > dihomo-γ–linolenic acid (C20:3 n-6). The values found for dihomo-γ–linolenic acid were close to 30% of those found for LO, whereas the values of γ-LA were about 50% of LO, for all of the studied cultivars (Table 4).

#### 2.2.4. Antioxidant Activity of the OLEs from Selected Cultivars

**Figure 1:**
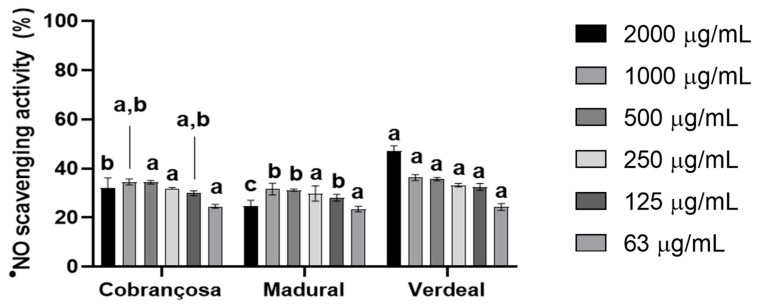
^●^NO scavenging activity of *O. europaea* leaf extracts. Three assays were performed (*n* = 3). For each tested concentration, different lowercase letters mean statistically significant differences at *p* < 0.05.

The antioxidant activity of hydroethanolic extracts from *O. europaea* Cobrançosa, Verdeal, and Madural was first determined spectrophotometrically at 517 nm; secondly, the FRAP method measured the reduction of the complex Fe^3 +^ -TPTZ read at 593 nm. Lastly, the anti-^●^NO scavenging power was determined according to the method described in Soares et al., 2021, at 560 nm [36]. The tested extracts showed similar antioxidant behavior against both 2,2-difenil-1-picril-hidrazil (DPPH^●^) and ferric ion (Fe^3+^)-ligand complexes. Their reducing power followed a waning order of *O. europaea* Madural > Verdeal > Cobrançosa. The DPPH^●^ radical scavenging activity exhibited by the *O. europaea* Madural and Verdeal extracts was better than their ferric reducing power: 19.9 ± 2.5 and 19.6 ± 2.4 mgTE/g sample, respectively (internal data). The least-powerful antioxidant extract was *O. europaea* Cobrançosa: 16.6 ± 0.5 mg AAE/g sample against FRAP and 13.6 ± 1.9 mgTE/g sample against DPPH^●^ (internal data). Overall, regarding the scavenging activity of each OLE, the IC_50_ by ^●^NO was attained for concentrations above 2000 µg/mL extract (Figure 1 and Table 5).

#### 2.2.5. Antimicrobial Activity of Selected Cultivars

The antimicrobial activity exhibited by diluted hydroethanolic leaf extracts from *O. europaea* Cobrançosa, *O. europaea* Verdeal, and *O. europaea* Madural was tested through agar diffusion assays against the following Gram-positive bacterial strains: *Staphylococcus aureus* (ATCC 25923), *Staphylococcus epidermidis* (NCTC 11047), and *Bacillus cereus* (ATCC 14579); and the following Gram-negative strains: *Pseudomonas aeruginosa* (ATCC 10145), *Salmonella* Enteritidis (ATCC 13076), and *Escherichia coli* (NCTC 9001), following an adapted protocol from the guidelines of the Clinical and Laboratory Standards Institute (CLSI).

Diluted extracts showed an overall superior efficacy against the tested Gram(−) strains compared to Gram(+), with the best results being those of the *O. europaea* Madural and Verdeal extracts against the Gram(−) *P. aeruginosa* (ATCC 10145) and the Gram(+) *B. cereus* (ATCC 14579). Despite the *O. europaea* Madural and Verdeal extracts’ broader antimicrobial activity against Gram(−) strains, the inhibition diameter of the Gram(+) *B. cereus* (ATCC 14579) was the most-important overall finding for both cultivars: 10.67 ± 0.61 mm and 11.30 ± 0.70 mm, respectively (Table 6), representing about 172.6% and 163.0% of the positive control’s inhibition. On the other hand, *O. europaea* Cobrançosa’s extract achieved higher efficacy against Gram(−) *S. Enteritis*, with a relative inhibition of 163.96% compared to the positive control. Regarding Gram(+) strains, the cultivar that showed a wider antimicrobial activity was *O. europaea* Verdeal, followed by *O. europaea* Cobrançosa and Madural. Only the leaves of *O. europaea* Verdeal showed activity against both *S. aureus* (ATCC 25923) and *B. cereus* (ATCC 14579), with a result of about 126.74% compared to the positive control. No selected cultivar displayed antimicrobial activity against either Gram(+) *S. epidermidis* (NCTC 11047) or Gram(−) *E. coli* (NCTC 9001). Overall, the results showed a higher antimicrobial efficacy of *O. europaea* Verdeal against *B. cereus* (ATCC 14579) *> P. aeruginosa* (ATCC 10145) *> S. aureus* (ATCC 25923), with inhibition zones of about 11.30 ± 0.70, 11.83 ± 1.01, and 12.38 ± 0.60 mm, respectively (Table 6). The extract of *O. europaea* Madural functioned better against *B. cereus* (ATCC 14579) *> P. aeruginosa* (ATCC 10145) > *Salmonella* Enteritidis (ATCC 13076), with inhibition zones of about 10.67 ± 0.61 mm, 11.44 ± 0.94 mm, and 13.54 ± 0.10 mm, respectively (Table 6). *O. europaea* Cobrançosa’s extract acted superiorly against *Salmonella* Enteritidis (ATCC 13076)* > P. aeruginosa* (ATCC 10145) *> B. cereus* (ATCC 14579), with the inhibition zones being about 11.57 ± 0.80 mm, 13.04 ± 0.53 mm, and 13.16 ± 3.97 mm, respectively (Table 6).

#### 2.2.6. Vitamin E Profile of Selected Cultivars

**Figure 2:**
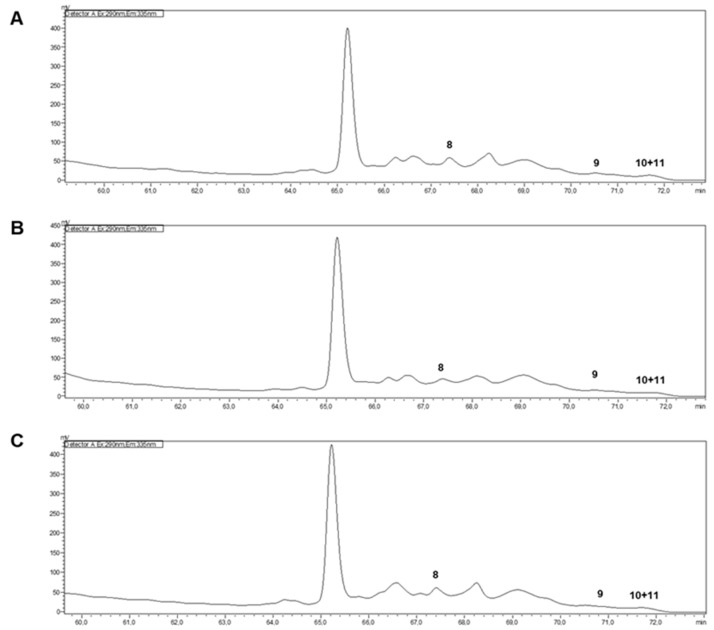
HPLC-FLD of hydroethanolic extracts of *O. europaea* leaves ((**A**)—*O. europaea* Cobrançosa; (**B**)—*O. europaea* Madural; (**C**)—*O. europaea* Verdeal; 8—α-tocopherol; 9—δ-tocopherol; 10 + 11—γ- and β-tocopherols).

The vitamin E profiles were determined through HPLC-FLD analysis of the hydroethanolic extracts from *O. europaea* Cobrançosa, *O. europaea* Verdeal, and *O. europaea* Madural. Very-low limits of detection (LOD) values were found for all tocopherols (α-tocopherol, δ-tocopherol, γ- and β-tocopherols (Figure 2)). So, no significant biological action could be attributed to E vitamers.

#### 2.2.7. Enzymatic Inhibition by Selected Cultivars

##### Acetylcholinesterase and Butyrylcholinesterase Inhibition

**Figure 3:**
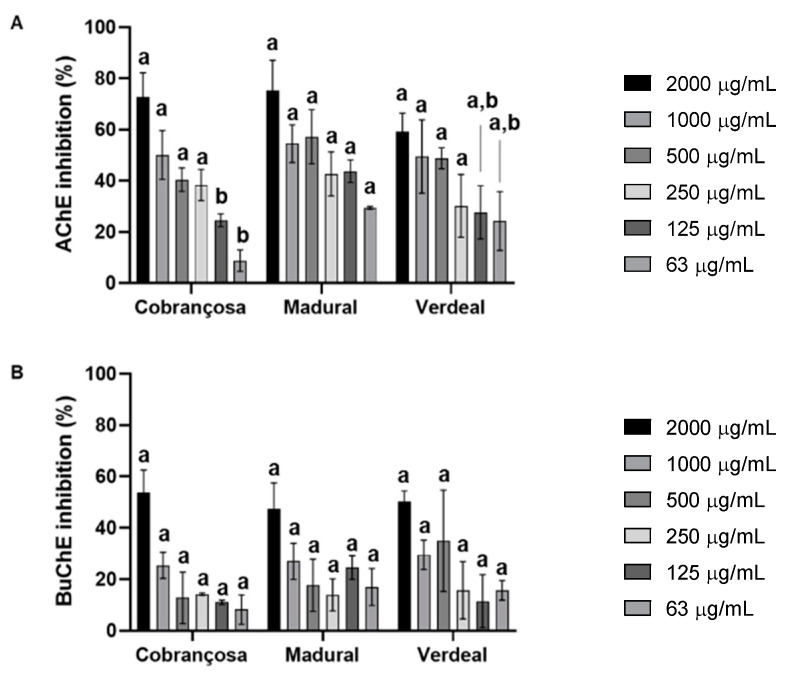
Acetylcholinesterase (AChE) (**A**) and butyrylcholinesterase (BuChE) (**B**) inhibition by extracts of *O. europaea* Cobrançosa, *O. europaea* Madural, *and O. europaea* Verdeal. Three assays were performed (*n* = 3). For each concentration tested, different lowercase letters mean statistically significant differences at *p* < 0.05.

The inhibition of the acetylcholinesterase (AChE) and butyrylcholinesterase (BuChE) enzymes by the hydroethanolic extracts was measured according to a modified Ellman method [37]. These results showed an overall higher inhibition of AChE compared to BuChE in the following order: *O. europaea* Madural > *O. europaea* Cobrançosa > *O. europaea* Verdeal, with IC_50_ values for AChE attained at 376.3 µg/mL, 995.5 µg/mL, and 1057.9 µg/mL, respectively (Figure 3 and Table 7).

##### Enzymatic Inhibition of Monoamine Oxidases A and B

**Figure 4:**
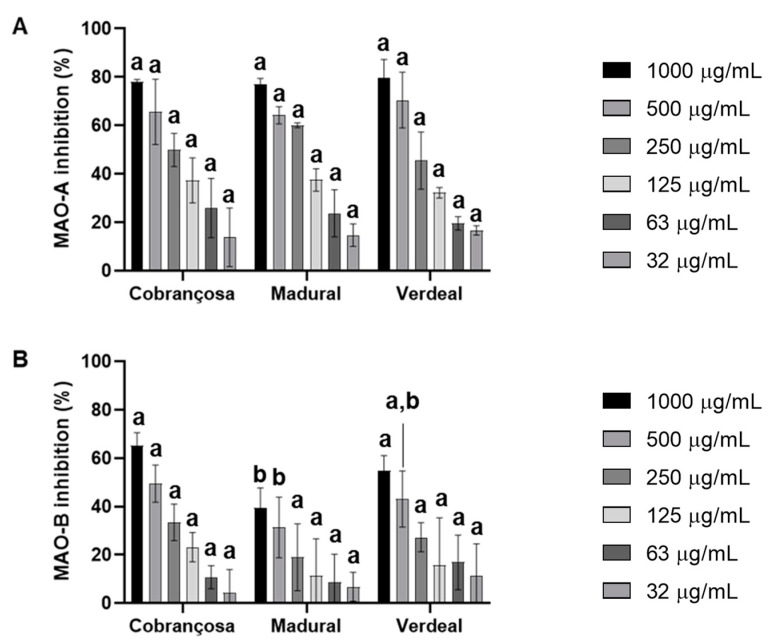
Monoamine oxidase A (MAO-A) (**A**) and B (MAO-B) (**B**) inhibition by extracts of *O. europaea* leaves. In total, three assays were performed (*n* = 3). For each tested concentration, different lowercase letters mean statistically significant differences at *p* < 0.05.

The inhibition of monoamine oxidases A (MAO-A) and B (MAO-B) by hydroethanolic extracts from *O. europaea* Cobrançosa, *O. europaea* Verdeal, and *O. europaea* was assessed based on the production of 4-hydroxyquinoline from kynuramine deamination, according to Soares et al., 2021 [37]. The results showed an overall higher inhibition of MAO-A enzymatic action by *O. europaea* Madural > Cobrançosa > Verdeal, with IC_50_ values registered at 194.1 µg/mL, 251.1 µg/mL, and 294.4 µg/mL, respectively (Figure 4 and Table 8).

##### Angiotensin-I-Converting Enzyme and Renin Inhibition

**Figure 5:**
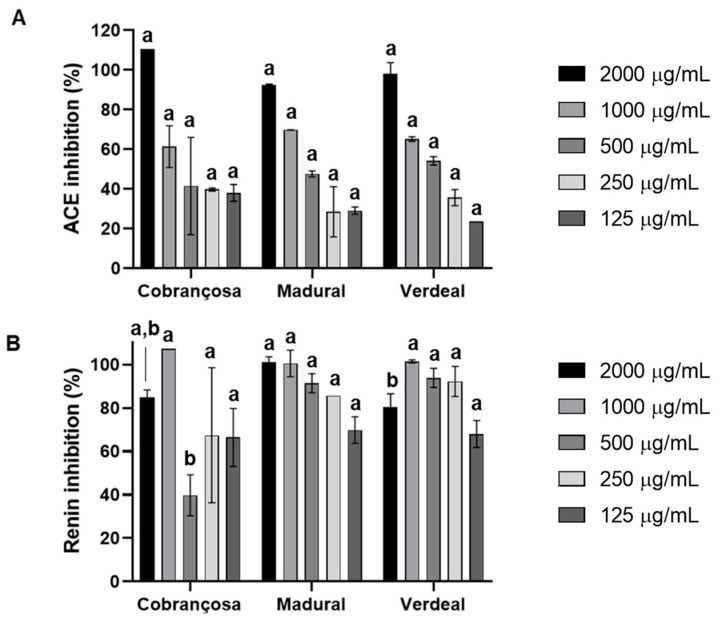
Angiotensin-converting enzyme (ACE) (**A**) and renin (**B**) inhibition by leaf extracts of *O. europaea* Cobrançosa, *O. europaea* Madural, *and O. europaea* Verdeal. In total, three assays were performed (*n* = 3). For each tested concentration, different lowercase letters mean statistically significant differences at *p* < 0.05.

Assessments of the inhibition of the angiotensin-I-converting enzyme (ACE) and renin by hydroethanolic extracts of *O. europaea* Cobrançosa, *O. europaea* Verdeal, and *O. europaea* Madural were performed with an ACE activity kit and renin assay kit, respectively, according to the manufacturer’s instructions. The results displayed an overall higher inhibition of renin for all *O. europaea* extracts. ACE inhibition followed an increasing power in the order of *O. europaea* Cobrançosa < *O. europaea* Madural < *O. europaea* Verdeal; IC_50_ values for ACE were reached at concentrations of 712.5 µg/mL, 553.2 µg/mL, and 442.4 µg/mL, respectively (Table 9 and Figure 5). On the other hand, the inhibitory power of the renin activity showed an increasing order as follows: *O. europaea* Madural < Verdeal < Cobrançosa—here, the IC_50_ readings didn’t allow a value discrimination for extract concentrations. Overall, the concentrations for inhibitory action against renin were all under 125 µg/mL (Figure 5 and Table 9).

## 3. Discussion

In the present study, the authors used leaf samples of cultivars that pose great importance for the economy of olive oil in Portugal. *O. europaea* Madural is one of the rarest olive varieties, mostly used for DOP (Protected Designation of Origin) olive oil. *O. europaea* Madural is known for generating a high yield of olive oil (22%), rich in linoleic acid, with organoleptic sensations varying from those of fresh herbs, apples, and nuts to comprise a lightly bitter and spicy taste [38]. This cultivar holds well in severe climates with a wide temperature range as well as in rough soils. *O. europaea* Cobrançosa originates from Alto Douro, Trás-os-Montes, next to the Spanish border. *O. europaea* Cobrançosa trees maintain a regular and large production, delivering an olive oil rich in polyphenols that offers a heightened resistance to oxidation and a balanced taste [38]. *O. europaea* Verdeal is a well-spread variety in Portugal due to its ability to adapt not only to the very-cold Trás-os-Montes but also to the very-hot Alentejo. Similarly to *O. europaea* Cobrançosa, the Verdeal cultivar yields large and regular olive harvests that account for a hefty portion of olive oil manufacture, holding particular features such as a thin texture, a persistent and marked fruity scent, and a quite bitter, spicy taste [38].

### 3.1. Mineral Analysis of the Selected Cultivars

The overall results determined that the major mineral components present in the selected cultivars were calcium (Ca), potassium (K), phosphorus (P), sulfur (S), and sodium (Na); Ca occupied the higher ranking for *O. europaea* Cobrançosa and Verdeal’s leaf extracts, whereas K came first in *O. europaea* Madural. These data are coherent with our previous study on *O. europaea* leaf extracts from an olive grove in another geographical area under different cultivation conditions [39]. Essentially, the inorganic analysis showed a composition in kg of leaves close to or above the daily recommended intake (DRI) values for the following components: Ca, Cr (except for the *O. europaea* Madural leaf extracts), Cu, Fe, K, Mg, Mn, Sr, Zn, and P, of which the Zn contents are also line with the last study [39]. Amidst metals currently considered essential for normal biofunctioning, our results identified Na, K, Mg, and Ca and block transition metal elements Mn, Fe, and Cu [29]. Chronic low body calcium levels in osteoporosis are usually counteracted through a balanced diet and calcium supplements, often associated with vitamin D and/or Mg [39]. A rich intake of Ca, vitamin D, and protein calls for higher levels of Mg, as well as hypomagnesemia, which may result in hypocalcemia and hypokalemia [40,41,42,43]. Malnutrition, obesity, and alcohol withdrawal might involve inorganic phosphate (Pi) or adenosine triphosphate (ATP) deficiencies, linked to bone diseases and myopathies, as well as the central and peripheral nervous system, for which the treatment requires a proper regulation of phosphorus. Manganese is implicated in several biological processes, with the literature describing manganese deficiencies as uncommon but holding a concern for the association of cancer susceptibility with low Mn-dependent superoxide dismutase activity. A vegetarian lifestyle prevents Mn deficiency though it might lead to iron deficiency [44], which in turn may lead to anemia, and copper build-up in the duodenal epithelium and liver [45]. Copper is predominantly found in the human brain, being absorbed in the intestine and excreted through bile [46]; moreover, it has been identified as a potential therapeutic target for obesity and nonalcoholic fatty liver disease (NAFLD), Alzheimer’s disease (AD), and Parkinson’s disease (PD), among others [39]. On the other hand, our results identified a particularly high value of As in the Verdeal extract of *O. europaea*, at 7 ± 0.03 mg/kg, which is concerning for the associated toxic effects. The latter’s inorganic study identified an As value of <0.25 mg/kg, much more inferior, probably due to differences already described before [39]. Several strategies for As removal from soil should be considered to avoid the intake of this element by the olive tree [47].The inorganic analysis in this work concedes us to propose OLE from selected cultivars as a practical and inexpensive source of mineral substrates to address the aforementioned disorders related to essential elements such as Ca, K, Mg, Mn, Fe, and Cu deficiencies. In particular, this study suggests *O. europaea* Madural’s OLE as a potential substrate candidate to address hypocalcemia and hypomagnesemia; *O. europaea* Verdeal’s leaf extract for hypokalemia and Mn deficiency, taking account the priority of removing As; and lastly, *O. europaea* Cobrançosa’s OLE to address hypocupremia and sideropenia.

### 3.2. Organic Assessment

#### 3.2.1. Nutritional Analysis and Fatty Acid Composition of the Selected Cultivars

Despite most of the samples showing a predominance of polyssacharide > protein > lipid > ash, the *O. europaea* Verdeal cultivar presented differently: polyssacharide > lipid > ash > protein. The lesser the water content, the higher the concentration of phenolic compounds becomes, and the more powerful the antioxidant activity is [48]; thus, in this work, *O. europaea* Verdeal’s leaves, with their slightly lower humidity values of 8.20 ± 0.35% (Table 2), could be the most useful substrate for retaining a higher-quality phenolic composition. Even so, this value is slightly lower compared to the minor water content values of 8.61 ± 0.12% and 8.43 ± 0.1% previously identified in the leaves of *O. europaea* Verdeal’s and Cobrançosa’s leaf sprouts [29]. The highest protein content was identified in *O. europaea* Madural’s leaves, at 6.43 ± 0.09% (Table 2), still comparatively lower than values from our previous study: 7.44 ± 0.02% in *O. europaea* Madural and 8.85 ± 0.20% in *O. europaea* Verdeal’s leaves [29]. The highest value of total fat content was shown by the *O. europaea* Cobrançosa leaves, at 5.03 ± 0.04% (Table 2). Despite the leaves from the remnant cultivars displaying a lesser amount of total lipidic content, being 4.80 ± 0.50% for *O. europaea* Verdeal and 4.50 ± 0.30% for *O. europaea* Madural, respectively (Table 2), these are still greater than the amount of total fat found in any sample of the previous study, where the upper value stands out at 4.01 ± 0.14% in the *O. europaea* Verdeal cultivar [29]. This might relate to differences between the cultivation methods, climate patterns, and soil composition concerning the two distinct harvest locations. Firstly, in Mirandela’s olive grove, the different cultivars were not planted separately by variety, as happened in the olive grove in Valpaços (previous study)—mix cultivation might favor wind cross-pollination, resulting in successful, cross-compatible combinations of olive cultivars. Plus, the higher temperatures of Mirandela (southern to Valpaços’s olive groves) might favor a higher lipidic but a lower protein content. Also, diverse cultivation procedures and soil properties may play differential factors in the leaf composition as well. With respect to the distribution of lipids, it follows a waning order as follows: SFAs > MUFAs > PUFAs for all tested cultivars. The SFA relative composition profiles for all of the samples showed to be as follows: palmitic acid (C16:0) > stearic acid (C18:0) > lignoceric acid (C24:0); the stearic acid value was about 25% of the palmitic acid value for all cultivars, while lignoceric acid was about 6% of the palmitic acid for *O. europaea* Cobrançosa and about 10% for both *O. europaea* Madural and Verdeal. FAs are involved in the following mechanisms: disruption of the electron transport chain by binding to electron carriers, the leakage of cell metabolites via cell lysis, inhibition of nutrient uptake, and the formation of peroxidation/auto-oxidation products, resulting in cell deactivation [49]. The aforementioned mechanisms could be acting behind the antibacterial activity of the *O. europaea* Cobrançosa and Verdeal extracts against *Salmonella* Enteritidis (ATCC 13076); at once, according to Table 4, these same cultivars also displayed the highest values for MUFAs. On the other hand, regarding the whole PUFA composition, the whole sum of omega-6 FAs is much higher than the total omega-3 FAs for all of the tested extracts, with an emphasis on omega-6 linoleic acid. Omega-3 FAs showed a poor trace presence for all tested extracts. Among the selected cultivars, PUFA contents varied in the following order: *O. europaea* Cobrançosa (39.9%) > *O. europaea* Verdeal (39.74%) > *O. europaea* Madural (37.87%) (Table 4); considering PUFAs as essential FAs for the human body, both *O. europaea* Madural’s and Verdeal’s leaf extracts surge as valuable substrates compared to those of *O. europaea* Cobrançosa. Regardless, *O. europaea* Verdeal’s extract, relative to other cultivars, showed a higher content of both PUFAs and MUFAs.

#### 3.2.2. TPC, TFC, and Antimicrobial Behavior of the Selected Cultivars

Several studies report that polyphenols derived from the olive tree are implicated in the relief and prevention of bioprocesses, such as through neurodegenerative, anti-hypertensive, and cardioprotective effects, whilst exhibiting anti-thrombotic, anti-inflammatory, antioxidant, anticancer, antimicrobial, and immune-protective properties [50,51]. The TPCs of the studied extracts were found to exhibit the following waning order: *O. europaea* Verdeal (59.30 ± 4.30 mgGAE/g sample) > Madural (48.90 ± 2.40 mgGAE/g sample) > Cobrançosa (37.90 ± 4.20 mgGAE/g sample) (Table 2), which are inferior to the values found for the same cultivars harvested from another geographical region in differential cultivation conditions, where *O. europaea* Cobrançosa (3.37 ± 0.25 mgGAE/g sample) > Verdeal (2.47 ± 0.2 mgGAE/g sample) > Madural (2.31 ± 0.22 mgGAE/g sample) [29]. Once again, *O. europaea* Verdeal’s leaf extract shows a relative higher level of TPC, which here is associated with a lower level of water content, suggesting it could be a potential source for extracting polyphenols of a high level of quality. On the other hand, *O. europaea* Verdeal’s leaves show the lowest level of protein and the highest As content among the three selected cultivars. For this, *O. europaea* Madural presents a better choice as a substrate for polyphenols, due to its TPC (Table 2) plus both, its high amount of protein (Table 2) and moderate water content (Table 2). With respect to the hypoglycemic and hypolipidemic properties already addressed in previous studies [52,53,54,55,56,57], the rich TPC in olive leaves, especially in the *O. europaea* Verdeal’s leaves of this work, makes them a possible tool for cardioprotective therapies as prophylactic and complementary measures, alongside their lower water content. The OLE of *O. europaea* Verdeal might also improve both insulin sensitivity and the secretory capacity of pancreatic β-cells, mimicking metformin effects in patients with type 2 diabetes mellitus (T2DM) [58]. So, if its As content is removed, this cultivar can also be suggested as a possible nutraceutical to regulate insulin resistance (IR) in developing endocrine disturbances. Among the TPCs found in the OLEs, hydroxytyrosol (HT) is referred to as the chief compound responsible for the biological properties of oleuropein (OLEP), mainly its high antioxidant potential [59]. HT has also been associated with innumerous favorable properties such as immuno-protective and antimicrobial activity [60,61], and anti-diabetic [62], anti-arrhythmic and cardioprotective [63], hypotensive and anti-atherosclerotic [64], anti-inflammatory [65], and antioxidant [66,67,68] effects. Besides HT, other phenolic compounds have shown to exhibit antibacterial effects against several strains of bacteria responsible for intestinal and respiratory infections in vitro [66,69,70,71], with promising results for applications as preservatives in food industry [23,62,72,73], in particular reducing the growth of Helicobacter pylori, which is associated with peptic ulcers and gastric cancer [66]. OLE polyphenols have also revealed antiviral and antiprotozoal activities [17,61,74,75,76]. The present study tested the inhibitory capacity of the OLEs from all three selected cultivars against Gram(+) strains—*S. aureus* (ATCC 25923)*, S. epidermidis* (NCTC 11047), *B. cereus* (ATCC 14579)—and Gram(−) strains—*E. coli* (NCTC 9001), *Salmonella* Enteritidis (ATCC 13076), and *P. aeruginosa* (ATCC 10145). Inhibition tests exposed an overall greater efficacy against the tested Gram (−) strains compared to the tested Gram(+) strains, with the particular best results being achieved by *O. europaea* Madural’s and Verdeal’s leaf extracts against the Gram (−) *P. aeruginosa* (ATCC 10145) and the Gram(+) *B. cereus* (ATCC 14579). Although both the *O. europaea* Madural and Verdeal varieties displayed a broader antimicrobial activity against Gram (−) strains, the best result attained for their overall antimicrobial activity was found for Gram(+) *B. cereus* (ATCC 14579), with inhibition diameters of 10.67 ± 0.61mm and 11.30 ± 0.70mm, which represent about 172.6% and 163.0% of relative microbial inhibition when compared to lactic acid (the positive control), respectively. On the other hand, the OLE of *O. europaea* Cobrançosa reached higher efficacy against Gram (−) *S. enteritis*, with a relative inhibition of about 163.96% compared to the positive control. Regarding Gram(+) strains, the cultivar that showed a wider antimicrobial activity was *O. europaea* Verdeal, followed by *O. europaea* Cobrançosa and *O. europaea* Madural. The leaves of *O. europaea* Verdeal only displayed activity against *S. aureus* (ATCC 25923) besides *B. cereus* (ATCC 14579), with a result of about 126.74% compared to lactic acid. No antimicrobial activity was found for any sample of leaves against either *S. epidermidis* (NCTC 11047) or *E. coli* (NCTC 9001). Overall, the results indicated a higher efficacy of the OLE of the *O. europaea* Verdeal cultivar against *B. cereus* (ATCC 14579) > *P. aeruginosa* (ATCC 10145) > *S. aureus* (ATCC 25923), with inhibition diameters of about 11.30 ± 0.70 mm, 11.83 ± 1.01 mm, and 12.38 ± 0.60 mm, respectively (Table 6). The OLE of the *O. europaea* Madural cultivar displayed a higher antimicrobial efficacy against *B. cereus* (ATCC 14579) *> P. aeruginosa* (ATCC 10145) *> Salmonella* Enteritidis (ATCC 13076), with inhibitory zones of about 10.67 ± 0.61 mm, 11.44 ± 0.94 mm, and 13.54 ± 0.10 mm, respectively (Table 6). Lastly, the efficacy of the OLE of *O. europaea* Cobrançosa was superior against *Salmonella* Enteritidis (ATCC 13076) *> P. aeruginosa* (ATCC 10145) *> B. cereus* (ATCC 14579), with inhibition diameters of about 11.57 ± 0.80 mm, 13.04 ± 0.53 mm, and 13.16 ± 3.97 mm, respectively (Table 6). It is known that on one hand, higher C-reactive protein and IL-6 levels have been associated more with Gram(−) bacteria than Gram(+) bacteria [77,78], inducing greater inflammation and thus raising concern regarding the treatment of Gram(−) sepsis. On the other hand, the SCOPE project (Surveillance and Control of Pathogens of Epidemiologic Importance) in 2000, found that Gram(+) bacteria accounted for 76% of infections in health-compromised individuals, compared to this being only 14% for Gram(−) microorganisms [79,80]. Adding these concerns to the worldwide issue regarding multidrug-resistant (MDR) microorganisms such as *B. cereus* (ATCC 14579), *S. aureus* (ATCC 25923), and *E. coli* (NCTC 9001), researchers keep looking for sustainable solutions to downplay the overuse of antibiotics in the medical field. Such solutions include designing pharmacological actives based on natural resources, with the olive leaf extract being considered a top source due to its wide range of antimicrobial activities. Silver nanoparticles have proved able to stop Gram(+) and Gram (−) bacteria populations and exhibit a wide range of antibacterial action, so some researchers tested Ag nanoparticles/attapulgite nanocomposites using an OLE with antioxidant and antibacterial activity [81]. These researchers confirmed this ability of olive leaf waste to be valorized into silver nanoparticles and to act upon MDR bacteria with inhibition zones occurring at higher concentrations (100 µg/mL) [82]. Moreover, the inhibitory activity against MDR bacteria by composite OLE AgNPs was superior to that of the standard antibiotic. The authors explain that OLE bioactive groups surrounded the surface of the AgNPs, leading to superior antioxidant, antimicrobial, and anticancer activities [82]. These studies did not identify the type of cultivar they employed to synthetize the NPs, but from our work, it is possible to propose all of the tested cultivars to create AgNPs against Gram(+) *B. cereus* (ATCC 14579), while *O. europaea* Verdeal’s leaves show the most adequate matrix for producing green AgNPs against Gram(+) *S. aureus* (ATCC 25923). Our results also suggest *O. europaea* Madural to be the most suitable extract against Gram (−) *P. aeruginosa* (ATCC 10145), while *O. europaea* Cobrançosa and Verdeal are shown to be more appropriate against Gram (−) *Salmonella* Enteritidis (ATCC 13076). Phenolic compounds such OLEP, TY, and HT evidenced immuno-protective and antimicrobial properties [25,83], wherein the anti-Gram(+) and anti-Gram(−) effect that has been most studied and credited to phenolics is membrane disruption. The hydroxyl groups in phenolics lead to electron delocalization, making them act as proton exchangers and promoting bacterial membrane disruption and so leading to the cell’s death [49]. Regarding this aspect, the results showed that *O. europaea* Madural’s extracts displayed the top concentration of HT, though very close to that of *O. europaea* Verdeal. Interestingly, the *O. europaea* Madural extract achieved a higher antimicrobial efficacy against *B. cereus* (ATCC 14579) and *P. aeruginosa* (ATCC 10145), followed by *O. europaea* Verdeal and Cobrançosa, mirroring the amount of HT found in them. Both *O. europaea* Madural’s and Verdeal’s extracts displayed a higher content of HT: 10.86 ± 0.88 and 10.64 ± 0.15 mg/g dried extract, respectively (Table 3), accounting for 38.76% and 46.12% of the whole phenolic composition, respectively (Table 3). The HT content alone is insufficient to explain the antimicrobial behavior of *O. europaea* Verdeal against *S. aureus* (ATCC 25923) as well as that of *O. europaea* Cobrançosa against *Salmonella* Enteritidis (ATCC 13076). The phenolic profile assessed in the present study for *O. europaea* Cobrançosa was as follows, in waning order: HT > apigenin derivative > apigenin-7-*O*-glucoside > luteolin-7-*O*-glucoside > verbascoside. The extract from *O. europaea* Madural showed a phenolic composition very close to *O. europaea* Cobrançosa’s extract, switching only the third position for luteolin-7-*O*-glucoside and the fourth position to apigenin-7-*O*-glucoside. Considering that both apigenin-7-*O*-glucoside and apigenin derivates have been reported to possess antimicrobial properties [84,85], their higher concentration in *O. europaea* Cobrançosa, associated with its higher content of MUFAs, could be speculated as a possible reason for it achieving the best result against *Salmonella* Enteritidis (ATCC 13076), but further studies are needed. The present study also revealed for *O. europaea* Verdeal a slightly different profile compared to both other extracts, following the waning order of HT > apigenin derivative > luteolin-7-*O*-glucoside > verbascoside > apigenin-7-*O*-glucoside. Interestingly, researchers have reported that luteolin-7-*O*-glucoside exhibits antimicrobial activity [86] against *S. aureus* (ATCC 25923) [87,88,89], like verbascoside [90,91], speculating a possible explanation for the differential positive activity of the *O. europaea* Verdeal extract against this microbial.

#### 3.2.3. Antioxidant Profile and Phenolic Composition of the Selected Cultivars

The TPCs of hydroethanolic extracts of *O. europaea* leaves showed to have relative higher presence in the following order: *O. europaea* Verdeal > *O. europaea* Madural > *O. europaea* Cobrançosa. The TFC results, however, showed another ranking: *O. europaea* Verdeal > *O. europaea* Cobrançosa > *O. europaea* Madural. Despite the highest TPC and TFC being shown by the leaf extract of *O. europaea* Verdeal, the results for the antioxidant activity of the OLEs showed *O. europaea* Madural > *O. europaea* Verdeal > *O. europaea* Cobrançosa regarding both FRAP and DPPH^●^-RSA reactions, mirroring the TPC and HT content results. *O. europaea* Madural displayed very close values for both reducing and oxidizing power—FRAP and DPPH^●^-RSA reactions, respectively. *O. europaea* Verdeal’s DPPH oxidizing power was close to that of *O. europaea* Madural, though the FRAP reducing power was comparatively lower, and for *O. europaea* Cobrançosa, the FRAP reducing power was higher comparatively to its DPPH oxidizing power. These differences in antioxidant and reducing behavior could be related to their relative composition of phenolic compounds. *O. europaea* Madural displayed the following profile for its antioxidant composition: HT > apigenin derivative > luteolin-7-*O*-glucoside > apigenin-7-*O*-glucoside, also associated to a stronger DPPH^●^-RSA activity, like *O. europaea* Verdeal’s extract. On the other hand, *O. europaea* Cobrançosa’s extract displayed a second higher content of apigenin derivative following the content of HT, possibly related to a higher activity of FRAP compared to DPPH^●^-RSA for the same cultivar. The highest content of HT and both glucosides of apigenin and luteolin could be behind this higher DPPH scavenging activity for the *O. europaea* Madural and *O. europaea* Verdeal extracts. *O. europaea* Cobrançosa’s leaves displayed the overall lowest value of TPC, at 37.90 ± 4.20 mg GAE/g sample, compared to the other two, despite displaying a TFC value very close to that of *O. europaea* Madural—30.40 and 29.10 mg ECE/g sample, respectively. Overall, the HT content showed to have a positive relation to the DPPH scavenging activity; this is in accord with review studies that have pointed out HT as a very significant bioactive compound mostly known for its high antioxidant efficiency, acting as a chain breaker by donating a hydrogen atom to peroxyl radicals (ROO^•^) by the o-dihydroxyphenyl moiety and thus replacing the ROO^•^ by the unreactive HTyr* radical [92,93,94]. A similar antioxidant behavior can be associated to the hydroxyl group at the ortho position at 3’ on the B ring in the flavonoid nucleus, making a signature of the antioxidant activity by flavonoids [95]. However, in the present study, the action upon DPPH seems more related to phenolic action rather than that of the flavonoids. If not, the higher content of specific flavonoid compounds such as apigenin derivates and apigenin-7-*O* and luteolin-7-*O* glucosides, rather than the TFC, might actually be more related to the reducing power from F^3+^ to Fe^2+^ of the olive leave extracts, in particular of *O. europaea* Madural. An additional antioxidant behavior of HT is associated to the activation of different cellular signaling pathways, such as the induction of phase II detoxifying enzymes through the activation of nuclear factor erythroid 2-related factor 2 (Nrf2) in distinct tissues [96]. On the other hand, HT’s ability to modulate the expression of several microRNAs (miRNAs) [97] makes it thus a potential indirect molecular target to consistently promote the expression of functionality-related genes with intervention in biological processes, portraying HT as a frontrunner for therapeutic and/or nutraceutical interventions. Furthermore, in vitro and in vivo data confirm the reduction in pro-inflammatory compounds such as inducible nitric oxide synthase (iNOS), cyclooxygenase-2 (COX-2), tumor necrosis factor-α (TNF-α), and interleukin (IL)-1β, as well as modulation of the immune response; however, the results for the scavenging activity of the olive leaf extracts against NO radicals were not conclusive [98]. Overall, with its hypocholesterolemic, anti-thrombotic, hypoglycemic properties, HT has been suggested to have therapeutic potential for the treatment of (1) atherosclerosis, acting as a potential mitochondria-targeting antioxidant in the inflamed endothelium, (2) postprandial lipidemia and hypercholesterolemia, with increases in endogenous vitamin C levels, and (3) platelet aggregation, with analogous results to acetylsalicylic acid. The antioxidant power of HT is also reflected in its anticarcinogenic potential, with positive effects in the prevention of several types of cancer [83,99,100,101,102]. Regarding its chemical properties that allow an easy formulation free of toxicity, HT has been highly regarded as a prime food supplement by the nutraceutical and food industries and so it is of key importance to potentiate the extraction of this phenolic compound from the olive leaf [61]. Concerning the premium choice, the *O. europaea* Madural leaf extract presents as a worthy nutraceutical due to its HT levels, confirming the invaluable multidimensional properties of olive leaves as a low-cost source of high-added-value phenolic compounds, as determined by other authors [30,31,51,99,103].

#### 3.2.4. Vitamin E Composition of the Selected Cultivars

The present study reveals a non-significant presence of tocopherols, an evident difference in relation to our previous study [29], where the OLEs’ tocopherol profiles varied in the waning order of *O. europaea* Verdeal (21.6 mg/100 g) > *O. europaea* Cobrançosa (18.79 ± 0.54 mg/100 g) > *O. europaea* Madural (12.03 ± 0.11 mg/100 g). In addition, from the overall content of tocopherols, it was also possible to identify α-T as the major isomer [29], with a waning order of 97.04% (*O. europaea* Verdeal) > 96.01% (*O. europaea* Madural) > 95.37% (*O. europaea* Cobrançosa). This difference may be due to both distinct cultivation procedures and the geographic location of the groves. The present study used samples of olive leaves collected in Mirandela within an orchard where the olive trees were distributed heterogeneously in the field, while in the previous study, the samples were harvested in Valpaços, where the olive groves were planted separately by cultivar. α-T is known for its antioxidant value in inhibiting lipid peroxidation, particularly within cell membranes rich in PUFAs, thus sustaining their integrity [104]. α-tocopherol is the core form of vitamin E, with the highest tissue concentration, but α- forms of both tocopherols and tocotrienols are considered the most metabolically active, with tocotrienols showing a higher antioxidant activity in in vivo systems [105]. The oral bioavailability of E vitamins is significantly limited as they are not recognized by the αT transfer protein (α-TTP), and recent studies suggest an alternative mechanism of intracellular transport of α-tocotrienol [106]. With respect to the identification of tocotrienols in OLEs, previous studies have not shown that there is a significant presence of this compound [29,107], but as different geographical regions induce diverse organic profiles, it would be interesting to broaden the study of tocotrienol levels in OLEs from diverse origins to understand if any of them possess the potential to be a source of both α- forms of tocopherols and tocotrienols and their possible use to mitigate neuro-inflammatory processes.

#### 3.2.5. Enzymatic Inhibition by the Selected Cultivars

Acetylcholinesterase (AChE) is responsible for the enzymatic cleavage of the neurotransmitter acetylcholine (ACh), for it is the main target for the mitigation of Alzheimer’s disease (AD), being present in both the central and peripheral nervous system and in muscular motor plaques [108]. Butyrylcholinesterase (BChE) is up-regulated in advanced AD, and it can be found in the brain, peripheral tissues, and in serum [108]. No drug has been approved to prevent neuronal cell loss in patients suffering from PD or AD once the “one protein, one target” strategy falls behind when addressing multifactorial neurodegenerative disorders. In contrast, restoring neurotransmitter levels by combinatorial inhibition of cholinesterases, monoamine oxidases, and adenosine A_2_A A receptors, to counteract oxidative stress and beta-amyloid plaque accumulation, would establish a therapeutically strong multitarget approach [109]. Latest studies have shown that butyrylcholinesterase (BuChE) inhibitors or double acetyl and butyryl cholinesterase inhibitors exhibit enhanced restorative effects on AD with less side effects than specific AChE inhibitors [108]. According to the literature, OLE reduces the activity of AChE and induces glutathione S-transferases in a dose–response manner [110,111]. Interestingly, the present study showed that all extracts could inhibit both AChE and BChE, with higher results against AChE: *O. europaea* Madural > *O. europaea* Verdeal > *O. europaea* Cobrançosa. Former studies have associated the role of specific compounds such as luteolin-7-*O*-glucoside to the enzymatic inhibition of AChE [89], which is coherent with the findings of a higher value of this flavone in *O. europaea* Madural compared to both other cultivars.

Monoamine oxidase (MAO) catalyzes the oxidative deamination of neurotransmitter monoamines and a variety of xenobiotic and dietary amines. Two MAO isoforms, namely MAO-A and MAO-B, are localized in the mitochondrial outer membrane and show different substrate specificities—MAO-A prefers serotonin, whereas MAO-B prefers phenylethylamine and benzylamine. Although most marketed MAO drugs are long-lasting irreversible inhibitors, reversible inhibitors have been developed to avoid prolonged irreversible effects: for example, moclobemide and safinamide, which are selective for MAO-A and MAO-B, respectively, and are prescribed as an antidepressant and for the treatment of PD, respectively [109,111,112]. Since both cholinesterases and monoamine oxidases are closely associated with AD and PD symptomatology and progression, they have been tackled simultaneously using several multifunctional ligands [109]. On one hand, several herbal extracts perform combined actions aimed at multiple receptors; on the other hand, the discovery of active natural products (i.e., secondary metabolites) can be either employed in clinical studies or function as important frames to design novel synthetic agents. Regarding the importance of cholinesterase and monoamine oxidase B for the treatment of AD and PD, innovative natural agents concomitant to novel biological activities present in known natural products have been explored in the last decade [111]. In the present study, an overall higher inhibition of MAO-A compared to MAO-B was observed, as follows: *O. europaea* Madural > *O. europaea* Cobrançosa > *O. europaea* Verdeal, wherein the IC_50_ for MAO-A was attained at concentrations of 194.1 µg/mL, 251.1 µg/mL, and 294.4 µg/mL, respectively (Table 8). These results are coherent with previous authors associating the inhibitory activity of MAO with specific flavones such as apigenins and luteolins [113]; however, further compound isolation studies of our samples are required to verify this premise. Still, the dual ability of the leaf extract from *O. europaea* Madural to inhibit AChE and MAOs surges as an interesting natural target for designing innovative pharmacological solutions to address AD and PD, or even depression.

Currently available ACE and renin inhibitors help to reduce the complications of hypertension; however, most patients require two or more anti-hypertensive medications to achieve their blood pressure (BP) goals (<140/90 mm Hg or <130/80 mm Hg for those with diabetes or chronic kidney disease), translating into an elevated risk of adverse drug reaction and a rise in medication costs [114]. An interesting alternative for upgrading efficacy while reducing therapeutic costs is the resource of potential herbal medicines, since their phytochemicals can either act directly on blood vessels through a vasorelaxant effect or indirectly through inhibition or stimulation of diverse systems, such as ACE, renin–angiotensin system (RAS), or diuretic activity [115]. The anti-hypertensive and cholesterol-lowering actions of olive leaves are well documented [21], as well as their hypotensive bioactives such as oleuropein, oleacein and oleanolic acid, flavonoids, alkaloids, tannins, and terpenoids [116,117]. OLE taken at 1000 mg daily effectively lowered systolic and diastolic blood pressures in subjects with stage-1 hypertension, with a comparable effect to a Captopril^®^ dose of 12.5–25 mg twice daily [118]. The present study showed an overall higher inhibition of renin activity compared to ACE with no possible dose distinction between OLEs of different cultivars. Regarding ACE inhibition, it follows an increasing order of *O. europaea* Cobrançosa< *O. europaea* Madural< *O. europaea* Verdeal, wherein the IC_50_ for ACE was attained at concentrations of 712.5 µg/mL, 553.2 µg/mL, and 442.4 µg/mL, respectively (Table 9). Considering the arsenium content in *O. europaea* Verdeal’s leaves, the *O. europaea* Madural leaf extract may be a good option as a source of natural compounds to reduce BP with it achieving the best inhibition of ACE and renin, revealing the best dual activity for BP modulation and so being an alternative to combinatory drug therapy.

## 4. Materials and Methods

### 4.1. Chemical Reagents

For the inorganic analyses, absolute ethanol was obtained from Fisher Chemical^®^ (Loughborough, England), and methanol, gallic acid, Folin–Ciocalteu reagent, sodium carbonate (Na_2_CO_3_), boron trifluoride (BF_3_), and 1,4-dioxane were purchased from Sigma (St. Louis, MO, USA). Nitric acid (HNO_3_), hydrogen peroxide (H_2_O_2_), Kjeldahl tablet catalysts, sulfuric acid, boric acid, potassium hydroxide (KOH), anhydrous sodium sulfate (Na_2_SO_4_), and *n*-hexane (HPLC grade) were obtained from Merck^®^ (Darmstadt, Germany). Tocol (2-methyl-2-(4,8,12-trimethyl-tridecyl) chroman-6-ol) was obtained from Matreya Inc.^®^ (State College, PA, USA). Vitamin E standards were from Calbiochem^®^ (La Jolla, CA, USA). Water was purified in a Milli-Q system^®^ (Millipore, Bedford, MA, USA). For the analyses of biological activities, we used Tris(hydroxymethyl)aminemethane (Tris), 5,5′-dithiobis(2-nitrobenzoic acid) (DTNB), acetylthiocholine iodide (ATCI), S-butyrylthiocholine iodide (BTCI), acetylcholinesterase (AChE) of Electrophorus electricus, butyrylcholinesterase (BuChE) of equine serum, bovine serum albumin (BSA), monoamine oxidase A (human recombinant MAO-A), monoamine oxidase B (human recombinant MAO-B), kynuramine, renin assay kit (MAK157), angiotensin-I-converting enzyme (ACE) activity assay kit (fluorometric) (CS0002), potassium phosphate monobasic (KH_2_PO_4_), potassium phosphate dibasic trihydrate (K_2_HPO_4_·3H_2_O), sodium nitroprusside dihydrate (SNP), sulfanilamide, naphthylethylenediamine dihydrochloride, ortho-phosphoric acid 85%, 2,2-diphenyl-1-picrylhydrazyl (DPPH), 6-hydroxy-2,5,7,8-tetramethylchroman-2-carboxylic acid (Trolox), 2,2′-azino-bis(3-ethylbenzothiazoline-6-sulfonic acid) (ABTS), potassium persulfate (K_2_O_8_S_2_), sodium carbonate, Folin–Ciocalteau reagent, gallic acid, boron trifluoride–methanol (BF_3_) at 14% methanol, butylated hydroxytoluene (BHT) (≥99%), and sodium nitrite were purchased from Sigma-Aldrich^®^ (St. Louis, MO, USA and Steinheim, Germany). Fatty acid methyl ester standard mixture (FAME) Supelco 37 was obtained from Supelco^®^ (Bellefonte, PA, USA). Epicatechin was obtained from Extrasynthèse^®^ (Genay, France). Magnesium chloride hexahydrate and sodium chloride were obtained from VWR^®^ (Leuven, Belgium). Sodium hydroxide and anhydrous sodium sulfate were obtained from Pronalab^®^ (Lisbon, Portugal). Dichloromethane and n-hexane (99%) from Merck^®^ (Darmstadt, Germany). Methanol from VWR Chemicals Prolabo^®^ (Fontenay-sous-Bois, France). Tridecanoic acid (C13:0) was obtained from Fluka^®^ (Switzerland) and sodium chloride (99.5%) was from Panreac^®^ (Barcelona, Spain). Aluminum chloride was purchased from Honeywell Fluka^®^ (Seelze, Germany). Standards for HPLC were purchased from Extrasynthèse^®^ (hydroxytyrosol, tyrosol, verbascoside, apigenin-7-*O*-glucoside, luteolin-7-*O*-glucoside), Fluka^®^ (caffeic acid), and Sigma-Aldrich^®^ (α-tocopherol, β-tocopherol, δ-tocopherol, and γ-tocopherol). PTFE 0.45 μm filters were purchased from VWR International^®^ (PA, USA). Ultrapure water (resistivity of 18.2 MΩ cm at 25 °C) was produced using a Simplicity 185 system (Millipore, Molsheim, France). The eluents used in the HPLC-DAD analyses were methanol Chromasolv for HPLC from Riedel-de Haën^®^ (Seelze, Germany) and formic acid from Carlo Erba^®^ (Val de Reuil, France), which were filtered through a 0.22 μm nylon membrane filter (Fioroni Filters, Ingré, France) using a vacuum pump (Dinko D-95, Barcelona, Spain) and degassed for 15 min in an ultrasonic bath (Sonorex Digital 10P, Bandelin DK 255P, Germany).

### 4.2. Sample Collection

**Figure 6:**
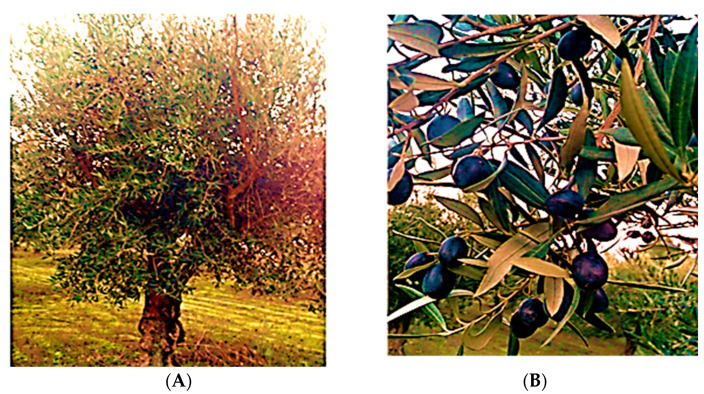
(**A**) Olive tree of *O. europaea* Madural. (**B**) Fruits and leaves of *O. europaea* Madural. Photos taken from the olive grove in Vale de Salgueiros, Mirandela, in May 2022.

**Figure 7:**
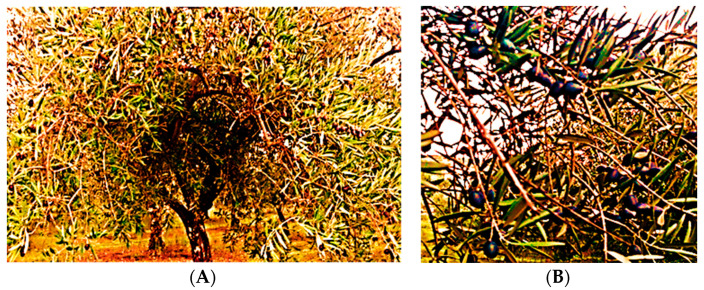
(**A**) Olive tree of *O. europaea* Cobrançosa. (**B**) Fruits and leaves of *O. europaea* Cobrançosa. Photos taken from the olive grove in Vale de Salgueiros, Mirandela, in May 2022.

**Figure 8:**
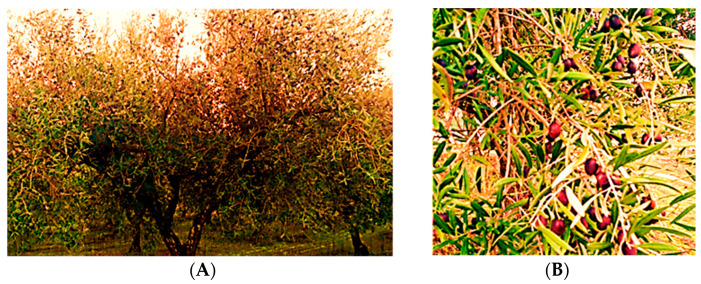
(**A**) Olive tree of *O. europaea* Verdeal. (**B**) Fruits and leaves of *O. europaea* Verdeal. Photos taken from the olive grove in Vale de Salgueiros, Mirandela, in May 2022.

The olive leaf samples were harvested from an olive grove in Vale Salgueiros, Mirandela region, during the month of May 2022. These samples belonged to three different cultivars: *O. europaea* Madural (Figure 6A,B)*, O. europaea* Cobrançosa (Figure 7A,B), and *O. europaea* Verdeal (Figure 8A,B), randomly mix-cultivated within the same grove. The olive grove’s coordinates in WGS84 are Lat: 41.579700 and Long: −7.241642. The main reason for the choice of these *O. europaea* cultivars is their strategic importance for obtaining the finest and most-desired organoleptic and physio-chemical properties in a premium olive oil with DOP (Protected Designation of Origin) excellency. All through the cycle of olive oil production, a bulky load of *O. europaea* leaves has to be disposed of, causing substantial costs for both olive farmers and oil manufacturers. Finding a way to upcycle these leaves based on the discriminated properties of each cultivar would not only create an economic and environmental value-adding route, but also a potential horizon for expansion in pharmaceutical and medical fields, food production, and preservation technologies, while respecting the basic principles of wise, good, and local established origins of raw material.

This production is embedded in a total area of 13.5 ha equivalently distributed with (1) 59.26% (8.0 ha) of *O. europaea* Cobrançosa, (2) 10.37% (1.4 ha) of *O. europaea* Madural, and (3) 30.37% (4.1 ha) of *O. europaea* Verdeal cultivars. On average, the allocation of olive trees/area is approximately 200 items/49 m^2^, being even denser in topographical planes. Soil mobilization was carried out through tillage. These cultivars were treated through the seasons with pesticides or herbicides properly certified for agroproduction, in coherence with an integrated protection program which allows the sustainable use of these products with reduced human health and environmental risks. This strategy promoted phytosanitary protection with the least-possible disturbance to agricultural and agroforestry ecosystems while encouraging natural mechanisms adverse towards plagues. According to our analysis of the leaves collected from the ground, the evidence suggested the use of a blend composed of (1) a calcium sulfate complex (Bordeaux mixture without copper), (2) Sprintplus (algae-based), and (3) mixed organic matter with soil revitalizers in the culture soil.

### 4.3. Sample Preparation and Extraction Procedure

Three varieties of *O. europaea* L. *folium* (*O. europaea* Cobrançosa, *O. europaea* Madural, and *O. europaea* Verdeal) were collected and ground in a mill (Retsch Knife Mill GRINDOMIX GM 200, Retsch, Haan, Germany). For inorganic evaluation, 0.2 g of each sample of milled dry leaf was digested by microwaves (MW) within a closed system at 170 °C using 1 mL of HNO_3_, 2 mL of H_2_O_2_, and 1 mL of H_2_O. After cooling, the vessel contents were transferred to volumetric flasks and the volume was increased to 25 mL by adding deionized water [39]. The samples for bioactive compound analysis were obtained by subjecting 0.60 g of milled dry olive leaves from each cultivar to an organic extraction procedure with 12 mL of an ethanol/water mixture (50/50, *v*/*v*) during 60 min at 55 °C. After extraction, the solvent was evaporated under reduced pressure [29]. The purpose of this work was to determine the presence and quantity of already-known compounds from previous studies [32,39] with these types of cultivars, for which no work of MS and HPLC has been performed for the characterization of their extracts.

### 4.4. Water Content

The assessment of humidity was determined in triplicate through infrared hygrometry readings at 105 °C using 1 ± 0.1 g of milled leaves from each sample (infrared balance, Scaltec model SMO01, Scaltec Instruments, Heiligenstadt, Germany).

### 4.5. Quantification of Inorganic Elements 

Mineral measurements of *O. europaea L. folium* were obtained using inductively coupled plasma mass spectroscopy (ICP-MS) on a Thermo ICP-MS X Series equipped with a Burgener nebulizer. This system functions with a plasma power at 1400 w, an argon flux of 13 mL/min, an auxiliary gas flux of 1 mL/min, and a sample flux of ~1mL/min. The tuning procedure was performed daily using a multielement solution (^6^Li, In, Ce, U: 10 µg/L each) and the response for oxides (^140^Ce^16^O/^140^Ce ratio) did not exceed 2%. External calibration was performed using a multielement standard solution in 1% nitric acid (*v*/*v*) at the following element concentration levels: 0, 0.2, 0.4, 1.0, 2.0, 5.0, 10, 50, and 100 µg/L for minor constituents, and 0, 0.02, 0.04, 0.1, 0.2, 0.5, 1, 5, and 10 mg/l for major elements. The internal standard (10.0 µg/L 115In) was added online. Each analysis was carried out in triplicate readings per solution sample [39].

### 4.6. Proximate Organic Composition

Proximate composition analyses of the three olive tree leaf varieties were performed in triplicate, according to AOAC (2000, 2005, 2006) methods [32,33,34,35]: dry matter (in an oven at 105 °C to constant weight; P Selecta, Barcelona, Spain); ash (incinerated at 500 °C for 6h in a muffle furnace; Nabertherm B-180, Lilienthal, Germany); protein quantitation via the Kjeldahl method (AOAC, 2000) using a digestor (DK6 digester, Velp Scientific, Usmate, Italy) followed by distillation (Keltec System 1002, Foss Tecator, Hilleroed, Denmark) and titration. Total lipids were extracted and quantified according to Folch et al., 1957 and using Folch solution (dichloromethane–methanol 2:1 *v/v* with 0.01% butylated hydroxytoluene—BHT) [35].

### 4.7. Total Phenolic Content (TPC)

TPC values were determined via colorimetric assays based on Folin–Ciocalteu reagent, which reacts with reduced phenols to produce a stable blue product at the end of the reaction. The reaction mixture consisted of 25 μL of the sample or standard solution, 75 μL of deionized water, and 25 μL of Folin–Ciocalteu reagent (50× dilution). After 6 min in the dark, 100 μL of Na_2_CO_3_ (75 g/L) was added. Absorbance was measured at 765 nm, after a 90 min incubation at room temperature. Calibration curves were calculated using gallic acid (GA). Each experiment was carried out in triplicate and the results were expressed as the gallic acid equivalent (GAE) per gram of sample [119].

### 4.8. Total Flavonoid Content (TFC)

TFC values were determined via colorimetric assays based on the formation of flavonoid–aluminum compounds [119]. The reaction mixture consisted of 25 μL of a diluted sample, 100 μL of ultrapure water and 10 μL of 5 g/100 mL NaNO_2_ solution. After 5 min, 15 μL of 10 g/100 mL AlCl_3_ solution was pipetted, and after 1 min, 50 μL of 1 mol/L NaOH was also added. The solution was well mixed, and absorbance was read at 510 nm. Calibration curves were prepared with epicatechin, and the results were expressed as milligrams of epicatechin equivalent (ECE) per gram of sample. The experiment was carried out in triplicate.

### 4.9. Phenolic and Tocopherol Profiles

Twenty microliters of each extract was analyzed (n = 3) on an analytical HPLC unit (Shimadzu) composed of a low-pressure quaternary pump (model LC-20AT), a degasser (model DGU-20A5R), an auto-sampler (model SIL-20AT), a column oven (model CTU-20AC), and a photodiode array detector (model SPD-M20A High-Performance Liquid Chromatography PDA Detector) at 25 °C. Compound separation was achieved with a C18 Spherisorb ODS2 (25.0 × 0.46 cm; 5 μm particle size) column from Waters (Ireland). The solvent system consisted of formic acid 5% (A) and methanol (B), starting with 5% B, and installing a gradient to obtain 15% B at 3 min, 25% B at 13 min, 30% B at 25 min, 35% B at 35 min, 45% B at 39 min, 45% B at 42 min, 55% B at 47 min, 75% B at 56 min, 100% B at 60 min, 100% B at 65 min, and 5% B at 66 min, stopping at 80 min. The solvent flow rate was 920 μL/min. Spectral data from all peaks were collected in the range of 200–600 nm, and chromatograms were recorded at 280, 320, 340, and 350 nm. Data were processed on LabSolutions software. Compounds were identified by comparing their retention times and UV–vis spectra with standards injected in the same conditions and/or by comparison with the literature [28,120]. External calibration curves were prepared to quantify the identified compounds in the samples, using six concentrations (n = 3, each concentration): hydroxytyrosol (y = 5.23 × 10^6^x + 1.33 × 10^4^; R = 0.9998), tyrosol (y = 5.63 × 10^7^x + 6.54 × 10^4^; R = 0.9998), caffeic acid (y = 1.34 × 10^8^x − 9.92 × 10^4^; R = 0.9997), verbascoside (y = 3.76 × 10^7^x + 3.19 × 10^4^; R = 0.9998), apigenin-7-*O*-glucoside (y = 5.97 × 10^7^x − 4.90 × 10^4^; R = 0.9998), and luteolin-7-*O*-glucoside (y = 5.98 × 10^7^x − 1.80 × 10^5^; R = 0.9995). Peak areas were recorded at 280 nm for hydroxytyrosol and tyrosol, at 320 nm for caffeic acid and verbascoside, at 340 nm for apigenin-7-*O*-glucoside and an apigenin derivative, and at 350 nm for luteolin-7-*O*-glucoside. The identified compounds were quantified with their corresponding standards, except the apigenin derivative, which was quantified as apigenin-7-*O*-glucoside. Tocopherol profiles were determined with the same runs performed for the phenolic profile but using the RF-20A-XS fluorescence detector. Calibration curves of α-tocopherol (y = 7.64 × 10^8^x + 3.17 × 10^5^; R = 0.9987), β-tocopherol (y = 1.16 × 10^9^x + 1.34 × 10^7^; R = 0.9912), γ-tocopherol (y = 1.04 × 10^9^x + 7.67 × 10^6^; R = 0.9965), and δ-tocopherol (y = 3.91 × 10^9^x + 1.75 × 10^7^; R = 0.9885) were prepared from 5 concentrations of standards (n = 3, each concentration). Each compound’s identity was assessed by comparing their retention times with their corresponding standards injected in the same conditions.

### 4.10. Lipid Profile

#### 4.10.1. Lipid Extraction

Around 2 g of the ground sample was introduced into a cellulose cartridge (cellulose extraction thimbles 25 × 60 mm) and lipid extraction was performed via Soxhlet (Soxtest Trade Raypa) with 50mL of a mixture of n-hexane–dichloromethane (50:50) for 7 h at 110 °C. The final extract was placed in a previously weighted glass tube and the solvent was evaporated on a rotatory evaporator (Rotavapor^®^ R-200; Buchi, Flawil, Switzerland) at 40 °C with vacuum. After 24 h in the desiccator, the tube was weighed [36].

#### 4.10.2. Determination of Fatty Acids 

The lipid residue previously obtained was re-diluted in 4 mL of n-hexane and 2 mL of dichloromethane. To 3 mL of these diluted extracted, we added 37.5 mg of activated carbon. After vortexing and centrifugation, 2 mL was taken and 5 mL of sodium methanolate solution (NaOMe) at 0.5 M was added. Following new vortex agitation, the samples were heated to 100 °C for 10 min and cooled for 5 min in ice. Then, 5 mL boron trifluoride-methanol was added, and the samples were again heated to 100 °C for 30 min and cooled in ice for 5 min. Then, 1 mL of n-hexane with butylated hydroxytoluene (BHT) at 0.02% was added. The tubes were shaken in a vortex, and 2 mL of sodium chloride was added and centrifuged for 10 min at 2500 rpm. The top layer was retrieved and dried with anhydrous sodium sulfate [37,121]. Then, 150 µL was taken and evaporated to dryness with nitrogen and finally re-diluted in 150 µL of n-hexane. Gas chromatography analyses were performed on Shimadzu GC- 2010 equipped with a flame ionization detector (FID) and a Shimadzu AOC-20i auto-injector. Separation of FAMEs was carried out on an Agilent^®^ J&W Cp-Sil 88 capillary column (60 m × 0.25 mm I.D., 0.20 µm) from Santa Clara, USA. The operating conditions were as follows: a split–splitless injector was used in split mode with a split ratio of 1:50; the injection volume of the sample was 1.0 µL; the injector and detector temperatures were kept at 250 °C and 260 °C, respectively; and the temperature program was as follows: initial temperature 100 °C for 5 min, increased at 3 °C/min to 183 °C and held for 3 min, raised at 0.5 °C/min to 195 °C held for 3 min, increased again at 0.5 °C/min to 200 °C, and finally raised at 2 °C/min to 220 °C held for 12.33 min. The carrier gas was He 30 mL/min and the detector gas flows were as follows: H_2_, 40 mL/min; air, 400 mL/min. Fatty acid methyl esters were identified by comparison with known standard mixtures (Sigma 47,885-U Supelco 37 Component FAME Mix, USA) and quantified using the software GCsolution for GC systems (Shimadzu), as described previously [122]. FA contents (expressed in %) were obtained for each fatty acid and total FA area ratio.

### 4.11. Antioxidant Activity

#### 4.11.1. DPPH^•^ Radical Scavenging Activity

The DPPH-RSA of samples was determined spectrophotometrically at 517 nm against the stable nitrogen radical DPPH radical. DPPH^•^ radical is reduced to the corresponding hydrazine when it reacts with hydrogen donors, such as an antioxidant. In this technique, samples (25 μL) were mixed with 200 μL fresh ethanolic solution of DPPH^•^ radical, 0.04 mg/mL. The mixture, vigorously shaken, was left to stand for 30 min in the dark (until stable absorption values were observed) [119]. Calibration curves were prepared with Trolox solutions and the results were expressed as the Trolox equivalent (TE) per gram of sample. The experiment was carried out in triplicate.

#### 4.11.2. FRAP Assay

The FRAP method corresponds to the reduction, at acid pH, of the complex Fe^3+^-TPTZ to Fe^2+^-TPTZ, which is blue at 593 nm. FRAP determinations were obtained by adding 20 μL of sample to 180 μL of FRAP reagent (Fe^3+^-2,4,6-Tri(2-pyridyl)-s-triazine), 0.833 mmol/L. The solution was incubated at 37 °C for 4 min for color development and the absorbance was measured at 593 nm [119]. Calibration curves were prepared with ascorbic acid (AA) solutions and the results were expressed as the ascorbic acid equivalent (AAE) per gram of sample. The experiment was carried out in triplicate.

#### 4.11.3. ^•^NO Scavenging Activity

The procedure adopted was previously described in Soares et al., 2021 [37]. One hundred microliters of sodium nitroprusside was incubated with 100μL of sample (2000, 1000, 500, 250, 125, 63 µg/mL) for 60 min, at room temperature, under light. After incubation, 100 μL of Griess reagent (composed of 1% sulfanilamide and 0.1% naphthylethylenediamine in 2% phosphoric acid) was added to each well. The mixture was incubated at room temperature for 10 min, and the absorbance was read at 560 nm (Biotek Synergy HT microplate reader). Phosphate buffer was used as negative control and 2% phosphoric acid, instead of the Griess reagent, was added to the blanks.

### 4.12. Enzyme Inhibition

#### 4.12.1. AChE and BuChE Inhibition

The inhibition of AChE and BuChE activity was measured according to a modified Ellman method previously reported [37]. Briefly, the extracts were dissolved in Tris-HCl buffer (50 mM, pH = 8) to prepare different dilutions (2000, 1000, 500, 250, 125, 63 µg/mL). Then, 25 µL of each extract dilution was added to the wells, plus 125 µL of DTNB reagent, 50 µL of buffer B (Tris-HCl buffer + 0.1% albumin), 25 µL of ATCI/BTCI, and 25 µL of 0.44 U/mL AChE solution or 0.40 U/mL BuChE solution. Slopes were calculated from the kinetic curve obtained at 405 nm and 37 °C in a total reaction time of 1 min 44 s. Blanks were prepared by substituting the enzymes for buffer B, and Tris-HCl buffer (instead of the extracts) was used as a negative control.

#### 4.12.2. MAO-A and MAO-B Inhibition

The MAO-A/B inhibition assay was based on the production of 4-hydroxyquinoline from kynuramine deamination, according to Soares et al., 2021 [37]. Six dilutions of each extract were prepared in 0.1 M KH_2_PO_4_/K_2_HPO_4_ (pH 7.4), namely, 1000, 500, 250, 125, 63, and 32 µg/mL. Two hundred and fifteen microliters of each dilution was mixed with 10 µL of 3.75 mM of knuramine. The reaction was started by adding 75 µL of 17 U/mL MAO-A or MAO-B solution. Negative controls (without extract) and blanks (without enzyme) were also prepared. The production of 4-hydroxyquinoline was determined by measuring the absorbance at 314 nm with a Synergy HT (Biotek Instruments) microplate reader during incubation at 37 °C for 70 min.

#### 4.12.3. Angiotensin Converting Enzyme (ACE) Inhibition

The ability of the extracts to inhibit ACE was measured with an angiotensin-I-converting enzyme (ACE) activity kit (Sigma-Aldrich, CS0002). Briefly, each well contained 50 μL of each extract dilution (2000, 1000, 500, 250, 125 µg/mL) or 50 μL of buffer solution (in case of the negative control), 20 μL of ACE solution (diluted 500-fold in assay buffer), and 50 μL of substrate (diluted 100-fold in assay buffer). Sample blanks were prepared by substituting the enzyme for buffer. Measurements were carried out at 37 °C, using Ex/Em = 360/40, 460/40, in kinetic mode, in 5 cycles for 5 min.

#### 4.12.4. Renin Inhibition

The ability of the extracts to inhibit renin was measured with a renin assay kit (Sigma-Aldrich, MAK157). Briefly, each well contained 50 μL of each extract dilution (2000, 1000, 500, 250, 125 µg/mL) or 50 μL of buffer solution (in case of negative control), 20 μL of renin solution (1 μg/mL), and 50 μL of substrate (diluted 100-fold in assay buffer). Sample blanks were prepared by substituting the enzyme for buffer. Measurements were performed at 37 °C, using Ex/Em = 485/20, 590/35, in kinetic mode, collecting data every five minutes for 60 min.

### 4.13. Antimicrobial Activity

#### 4.13.1. Microorganisms and Culture Conditions

The antimicrobial activity of the olive tree leaf extracts was assessed against the following Gram-positive bacterial strains: *Staphylococcus aureus* (ATCC 25923), *Staphylococcus epidermidis* (NCTC 11047), and *Bacillus cereus* (ATCC 14579); and the following Gram-negative strains: *Pseudomonas aeruginosa* (ATCC 10145), *Salmonella* Enteritidis (ATCC 13076), and *Escherichia coli* (NCTC 9001). Bacterial strains were stored at −80 °C in glycerol–water (15:75, v:v). Prior to running the experiments, active cultures were grown in sterile Mueller–Hinton broth (MHB) at 37 °C, overnight. Then, an aliquot from each culture was transferred to fresh MHB and properly diluted to achieve an optical density of 0.09–0.11, measured at 600 nm (Clinical and Laboratory Standards Institute, 2012), corresponding to the 0.5 MacFarland standard to reach 1–2 × 10^8^ colony formation units (CFUs) for further assays.

#### 4.13.2. Agar Diffusion Assay

This assay followed the adapted protocol from the CLSI guidelines (Clinical and Laboratory Standards Institute, 2012). Briefly, 5 mL of each extract was evaporated until dryness under a nitrogen flow at 25 °C to minimize oxidation. The remaining residue was dissolved in 2 mL of DMSO, and filter-sterilized through 0.45 μm pore-size syringe filters. Later, 100 μL of such suspensions for every microorganism were seeded in Petri dishes containing Mueller–Hinton II agar and spread with sterile swabs. Plates were divided into four sections, including 15 μL of each tested extract (test section), DMSO (negative control section), and 40% lactic acid (positive control section). Once cultivated, the plates were incubated at 37 °C for 24 h. Growth inhibition was quantified in inhibition circular zones, and their diameters were measured using a digital caliper rule. Triplicate plates were assessed for each microorganism.

### 4.14. Statistical Analysis

Statistical analysis was performed using IBM SPSS Statistics (v. 26 for Windows, IBM Corp., Armonk, 241 NY, USA). The evaluation of statistical significance was determined by ANOVA and Tukey’s HSD to assess significant differences between the samples at a 5% significance level.

## 5. Conclusions

The inorganic profile of OLEs from different cultivars makes them a practical and inexpensive source of mineral substrates for addressing disorders related to deficiencies of essential elements such as Ca, K, Mg, Mn, Fe, and Cu, particularly proposing *O. europaea* Madural’s OLE to address hypocalcemia and hypomagnesemia; Verdeal’s for hypokalemia and for Mn deficiency, if its As is properly removed; and *O. europaea* Cobrançosa’s to address hypocupremia and sideropenia. The extract from *O. europaea* Madural’s leaves could be the most useful one for retaining a high-quality phenolic composition given the water content and TPC, making it a possible natural matrix for (1) cardioprotective therapies as a prophylactic and treatment agent, and (2) improving both the insulin sensitivity and (3) secretory capacity of pancreatic β-cells, thus mimicking metformin’s effects in patients with T2DM. Considering the variability patterns and differences in the parameters analyzed for the chemical profile of each OLE, it seems that the phenolic compounds—particularly HT and flavones as apigenin derivatives—are the key compounds responsible for the antimicrobial activity described. Nevertheless, other components such as the total composition of FAs such as MUFAs and PUFAs are possible synergistic factors, along with phenolics and flavonoids acting against bacteria together rather than acting solo. *O. europaea* Madural and Cobrançosa can be proposed as possible materials for formulating green and safe AgNPs to be used as antioxidants and as an alternative antibiotic against some MDR microorganisms in the future, since they are non-toxic, environmentally friendly, and affordable. In particular, the higher TPC and TFC of *O. europaea* Verdeal’s leaf extract makes it valuable against Gram(+) *S. aureus* (ATCC 25923), while *O. europaea* Madural’s leaves, rich in HT, showed the highest activity against the Gram-negative *P. aeruginosa* (ATCC 10145), and MUFA-rich *O. europaea* Cobrançosa showed to be more effective against Salmonella Enteritidis (ATCC 13076). Given its higher capacity to inhibit both AChE, BuChE, and MAO-A, *O. europaea* Madural’s leaf extract may be proposed as a natural multitarget treatment for AD, PD, and depression. Moreover, due to it lacking the arsenic toxicity present in the samples of *O. europaea* Verdeal and its favorable inhibition of both renin and ACE, *O. europaea* Madural’s leaf extract exhibits the best dual activity for BP modulation and so presents an alternative for combinatory drug therapy. This work confirms the multifaceted ability of olive leaves to be regarded as an olive tree byproduct to be exploited as a low-cost source of high-added-value phenolic compounds, displaying a variable profile of nutritional and biochemical criteria when changing parameters such as the orchard location, climate, and type of cultivation. It is known that *Olea europaea* L. is a wind-pollinated crop with a diverged bearing pattern [123]. Fruit and leaf features can be improved through numerous strategies to define the most successful compatible combinations of cultivars. A mixed cultivation of different varieties within the same area might lead to naturally selected crossbreeding, favoring certain properties. So, certainly, the dissimilarity in findings for the studied parameters between the present and the previous studies might be due to both geographic and climatic diversity, but also, to some degree, modification in the crop genotype. For verifying this influence on the newfound parameters’ variance, it would be recommended to follow this work with new studies regarding seed paternity analyses/genetic surveys of leaf samples, planned, for example, on microsatellite markers specified for the identification of potential pollen donors from the three cultivars that have shared this mixed olive orchard for over 60 consecutive years.

## Figures and Tables

**Table 1:** Mineral analysis of leaf samples of *O. europaea*. Study conducted with leaves extracted from three varieties of olive tree: *O. europaea* Cobrançosa, *O. europaea* Madural, and *O. europaea* Verdeal cultivars. These cultivars grew up randomly distributed in the same geographical area of Vale de Salgueiros in the Mirandela region of the North of Portugal.

	Cultivar	Units	*O. europaea* Cobrançosa	*O. europaea* Madural	*O. europaea* Verdeal	DRIs—Male (31–50 y.o.) **
Elements						
**As**		mg/kg	<3.0 ± 0.03	<3.0± 0.03	7 ± 0.03	N/A
**Ba**		mg/kg	18 ± 0.05	13 ± 0.05	12 ± 0.05	N/A
**Ca**		g/Kg	11 ± 0.05	12 ± 0.05	11 ± 0.05	1.00 g/d
**Cd**		mg/kg	<0.25	<0.25	<0.25	N/A
**Cr**		mg/kg	0.65 ± 0.03	<0.5	0.72 ± 0.03	35 mg/d
**Cu**		mg/kg	12 ± 0.05	5.6 ± 0.03	6.5 ± 0.03	0.90 mg/d
**Fe**		mg/kg	104 ± 0.05	91 ± 0.05	82 ± 0.05	8 mg/d
**K**		g/Kg	9.2 ± 0.03	7.1 ± 0.03	13 ± 0.05	3.40 g/d
**Mg**		g/Kg	1.2 ± 0.03	1.7 ± 0.03	1.4 ± 0.03	0.42 g/d
**Mn**		mg/kg	47 ± 0.05	46 ± 0.05	56 ± 0.05	2.3 mg/d
**Na**		g/Kg	0.05 ± 0.03	0.04 ± 0.03	0.07 ± 0.03	1.5 g/d
**Pb**		mg/kg	<2.5	<2.5	<2.5	N/A
**Se**		mg/kg	<2.5	<2.5	<2.5	0.055 mg/d
**Sr**		mg/kg	95 ± 0.05	101 ± 0.05	103 ± 0.05	14.0 mg/d
**Zn**		mg/kg	12 ± 0.05	17 ± 0.05	17 ± 0.05	11.0 mg/d
**Mo**		mg/kg	<3.0	<3.0	<3.0	0.045 mg/d
**S**		g/Kg	2.2 ± 0.03	0.37 ± 0.03	1.5 ± 0.03	N/A
**P**		g/Kg	1.3 ± 0.03	1.5 ± 0.03	1.5 ± 0.03	0.70 g/d

** DRIs (daily recommended intakes)—https://nap.nationalacademies.org/read/11537/chapter/59#536 (accessed on 7 December 2023).

**Table 2:** Proximate composition, total phenolic content (TPC), and total flavonoid content (TFC) of *O. europaea* leaf extracts.

	Component	Ash (%)	Lipid (%)	Protein (%)	Water (%)	TPC (mgGAE/g Sample)	TFC (mgECE/g Sample)
Cultivar							
***O. europaea* Cobrançosa**		4.34 ± 0.30 ^a^	5.03 ± 0.04 ^a^	6.15 ± 0.12 ^b^	8.86 ± 0.15 ^a^	37.90 ± 4.20 ^c^	30.40 ± 0.80 ^b^
***O. europaea* Madural**		4.42 ± 0.14 ^a^	4.50 ± 0.30 ^a^	6.43 ± 0.09 ^a^	8.70 ± 0.17 ^a,b^	48.90 ± 2.40 ^b^	29.10 ± 0.50 ^b^
***O. europaea* Verdeal**		4.79 ± 0.04 ^a^	4.80 ± 0.50 ^a^	3.81 ± 0.02 ^c^	8.20 ± 0.35 ^b^	59.30 ± 4.30 ^a^	53.70 ± 5.20 ^a^

GAE—gallic acid equivalent; ECE—epicatechin equivalent. Results are expressed as mean ± standard deviation of three determinations (*n* = 3). For each column/row, different superscript letters mean statistically significant differences at *p* < 0.05.

**Table 3:** Phenolic composition of the hydroethanolic extracts of the selected *O. europaea* leaves.

	Parameter	RT (min)	ʎmax (nm)	Cultivar (mg/g Dried Extract)		
Compounds				*O. europaea* Cobrançosa	*O. europaea* Madural	*O. europaea* Verdeal
**Hydroxytyrosol**		9.70	276	7.78 ± 2.00 ^a^	10.86 ± 0.88 ^a^	10.64 ± 0.15 ^a^
**Tyrosol**		12.50	280	0.13 ± 0.04 ^b^	0.27 ± 0.05 ^a^	0.20 ± <0.01 ^a,b^
**Caffeic acid**		18.26	250, 290sh, 323	0.19 ± 0.03 ^a^	0.20 ± 0.01 ^a^	0.21 ± 0.03 ^a^
**Verbascoside**		32.23	253, 296sh, 331	2.30 ± 0.16 ^a^	2.31 ± 0.04 ^a^	2.40 ± 0.43 ^a^
**Luteolin-7-*O*-glucoside**		42.44	257, 267sh, 348	2.72 ± 0.01 ^b^	4.27 ± <0.01 ^a^	2.69 ± 0.03 ^b^
**Apigenin-7-*O*-glucoside**		46.07	267, 336	2.91 ± 0.07 ^b^	3.82 ± 0.22 ^a^	2.01 ± 0.09 ^c^
**Apigenin derivative**		48.60	268, 338	5.03 ± 0.11 ^b^	6.32 ± 0.29 ^a^	4.92 ± 0.10 ^b^
**Total**				**21.06**	**28.05**	**23.07**

^ab^ Results are expressed as mean ± standard deviation for three assessments (*n* = 3). For each row, different superscript letters mean statistically significant differences at *p* < 0.05.

**Table 4:** Fatty acid composition (values expressed as % fatty acid ± SD).

	Cultivars	*O. europaea* Cobrançosa	*O. europaea* Madural	*O. europaea* Verdeal
FAs				
**SFA**				
C4:0		-	-	-
C6:0		-	-	-
C8:0		-	-	-
C10:0		-	-	-
C11:0		-	-	-
C12:0		0.347% ± 0.007%	0.28% ± 0.02%	0.24% ± 0.01%
C13:0		-	-	-
C14:0		2.48% ± 0.07%	1.94% ± 0.05%	2.85% ± 0.06%
C15:0		0.24% ± 0.01%	0.308% ± 0.006%	0.27% ± 0.01%
C16:0		33.6% ± 0.1%	31.3% ± 0.2%	30.9% ± 0.6%
C17:0		0.57% ± 0.03%	0.36% ± 0.02%	0.49% ± 0.02%
C18:0		8.15% ± 0.03%	8.8% ± 0.6%	7.0% ± 0.2%
C20:0		-	-	-
C21:0		0.34% ± 0.02%	0.47% ± 0.02%	0.53% ± 0.02%
C22:0		-	-	-
C23:0		0.51% ± 0.04%	0.80% ± 0.04%	0.38% ± 0.02%
C24:0		2.4% ± 0.2%	3.3% ± 0.3%	3.04% ± 0.03%
**MUFA**				
C14:1 n-5		-	-	-
C15:1 n-5		-	-	-
C16:1 n-7		1.18% ± 0.02%	0.57% ± 0.03%	0.48% ± 0.01%
C17:1 n-7		-	-	-
C18:1 n-9 t		-	-	-
C18:1 n-9 c		25.49% ± 0.09%	19.7% ± 0.3%	21.7% ± 0.2%
C20:1n-9		13.3% ± 0.1%	17.6% ± 0.2%	17.6% ± 0.2%
C22:1 n-9		-	-	-
C24:1 n-9		-	-	-
**PUFA**				
C18:2 n-6 t		-	-	-
C18:2 n-6 c		6.05% ± 0.04%	8.5% ± 0.2%	8.0% ± 0.1%
C18:3 n-3		0.52% ± 0.02%	0.622% ± 0.005%	0.71% ± 0.04%
C18:3 n-6		2.93% ± 0.03%	3.8% ± 0.1%	3.19% ± 0.10%
C20:2 n-6		-	-	-
C20:3 n-3		-	-	-
C20:3 n-6		2.01% ± 0.04%	2.3% ± 0.2%	2.3% ± 0.1%
C20:4 n-6		-	-	-
C20:5 n-3		-	-	-
C22:2 n-6		-	-	-
C22:6 n-3		-	0.44% ± 0.02%	0.40% ± 0.02%
ΣSFA		48.6%	46.71%	45.75%
ΣMUFA		39.9%	37.87%	39.74%
ΣPUFA		11.5%	15.42%	14.51%
Σω3		0.5%	0.84%	1.10%
Σω6		11.0%	14.58%	13.41%

**Table 5:** Antioxidant activity of hydroethanolic extracts of *O. europaea* leaves.

	Antioxidant Activity	DPPH^●^-RSA (ug/mL)	FRAP (mg AAE/g Sample)	^●^NO (IC_50_, µg/mL)
Cultivar				
*O. europaea Cobrançosa*		685.2 ±34.4 ^b^	16.6 ± 0.5 ^b^	>2000 (32.2% inhibition at 2000 µg/mL)
*O. europaea Madural*		490.8 ± 24.5 ^a^	19.1 ± 0.6 ^a^	>2000 (24.8% inhibition at 2000 µg/mL)
*O. europaea Verdeal*		217.2 ± 10.9 a	17.0 ± 0.01 b	>2000 (47.3% inhibition at 2000 µg/mL)

RSA—radical scavenging activity; FRAP—ferric reducing antioxidant power assay; TE—Trolox equivalent; AAE—ascorbic acid equivalent. Results are expressed as mean ± standard deviation of three determinations (*n* = 3). For each column, different superscript letters mean statistically significant differences at *p* < 0.05.

**Table 6:** Antimicrobial activity of *Olea europaea* extracts at 20 mg/mL, positive control (lactic acid), and negative control (DMSO).

*CULTIVAR*	Inhibition Zone (mm)					
	Gram(+)			Gram(−)		
	*S. aureus* (ATCC 25923)	*S. epidermidis* (NCTC 11047)	*B. cereus* (ATCC 14579)	*E. coli* (NCTC 9001)	*Salmonella* Enteritidis (ATCC 13076)	*P. aeruginosa* (ATCC 10145)
*O. europaea Cobrançosa*	0	0	13.16 ± 3.97 ^a,b^	0	11.57 ± 0.80 ^b^	13.04 ± 0.53 ^b^
*O. europaea Madural*	0	0	10.67 ± 0.61 ^b^	0	13.54 ± 0.10 ^b^	11.44 ± 0.94 ^b^
*O. europaea Verdeal*	12.38 ± 0.60 ^a^	0	11.30 ± 0.70 ^b^	0	12.67 ± 0.62 ^b^	11.83 ± 1.01 ^b^
*Lactic acid*	15.69 ± 2.07 ^a^	22.24 ± 1.19	18.42 ± 0.57 ^a^	17.67 ± 1.11	18.97 ± 1.40 ^a^	19.74 ± 3.61 ^a^
*DMSO*	0	0	0	0	0	0

Results are expressed as mean ± standard deviation of three determinations (*n* = 3). For each column, different superscript letters mean statistically significant differences at *p* < 0.05.

**Table 7:** IC_50_ values for AChE and BuChE inhibition by the *O. europaea* extracts from Cobrançosa, Madural, and Verdeal cultivars.

Sample	AChE—IC_50_ (µg/mL)	BuChE—IC_50_ (µg/mL)
***O. europaea* Cobrançosa**	995.5	1869.6
***O. europaea* Madural**	376.3	>2000 (47.4% inhibition at 2000 µg/mL)
***O. europaea* Verdeal**	1057.9	1987.1

**Table 8:** IC_50_ values for the inhibition of MAO-A and MAO-B by the extracts of *O. europaea* Cobrançosa, *O. europaea* Madural, and *O. europaea* Verdeal.

Sample	MAO-A—IC_50_ (µg/mL)	MAO-B—IC_50_ (µg/mL)
***O. europaea* Cobrançosa**	251.1	516.1
***O. europaea* Madural**	194.1	>1000 (36.0% at 1000 µg/mL)
***O. europaea* Verdeal**	294.4	792.1

**Table 9:** IC_50_ values for the inhibition of ACE and renin by leaf extracts from *O. europaea* Cobrançosa, *O. europaea* Madural, and *O. europaea* Verdeal.

Sample	ACE—IC_50_ (µg/mL)	Renin—IC_50_ (µg/mL)
***O. europaea* Cobrançosa**	712.5	<125.0 (66.4% inhibition at 125 µg/mL)
***O. europaea* Madural**	553.2	<125.0 (69.80% inhibition at 125 µg/mL)
***O. europaea* Verdeal**	442.4	<125.0 (68.01% inhibition at 125 µg/mL)

## Data Availability

The original contributions presented in this study are included in this article. Further inquiries can be directed to the corresponding author.
